# Bioinspired Theranostic Coordination Polymer Nanoparticles for Intranasal Dopamine Replacement in Parkinson’s Disease

**DOI:** 10.1021/acsnano.1c00453

**Published:** 2021-04-22

**Authors:** Javier García-Pardo, Fernando Novio, Fabiana Nador, Ivana Cavaliere, Salvio Suárez-García, Silvia Lope-Piedrafita, Ana Paula Candiota, Jordi Romero-Gimenez, Beatriz Rodríguez-Galván, Jordi Bové, Miquel Vila, Julia Lorenzo, Daniel Ruiz-Molina

**Affiliations:** † Catalan Institute of Nanoscience and Nanotechnology (ICN2), CSIC and BIST, Campus UAB, 08193 Bellaterra, Barcelona, Spain; ‡ Institut de Biotecnologia i de Biomedicina (IBB), Universitat Autònoma de Barcelona, 08193 Bellaterra, Barcelona, Spain; § Departament de Bioquímica i Biologia Molecular, Unitat de Bioquímica de Biociències, Edifici C, Universitat Autònoma de Barcelona, 08193 Cerdanyola del Vallès, Spain; ∥ Departament de Química, Universitat Autònoma de Barcelona (UAB), Campus UAB, 08193 Cerdanyola del Vallès, Barcelona, Spain; ⊥ Centro de Investigacion Biomédica en Red en Bioingeniería, Biomateriales y Nanomedicina (CIBER-BBN), 08193 Cerdanyola del Vallés, Spain; ^#^Servei de Ressonància Magnètica Nuclear, ^∇^Institut de Neurociències, Departament de Bioquímica i Biologia Molecular, Universitat Autònoma de Barcelona, 08193 Cerdanyola del Vallès, Spain; □ Neurodegenerative Diseases Research Group, Vall d’Hebron Research Institute (VHIR)-Center for Networked Biomedical Research on Neurodegenerative Diseases (CIBERNED), Edifici Collserola Hospital Universitari Vall d’Hebron, Passeig de la Vall d’Hebron, 129, 08035 Barcelona, Spain; ■ ICREA-Institució Catalana de Recerca i Estudis Avancats, 08010 Barcelona, Spain

**Keywords:** neuromelanin, dopamine, Parkinson’s disease, neurodegeneration, coordination polymers

## Abstract

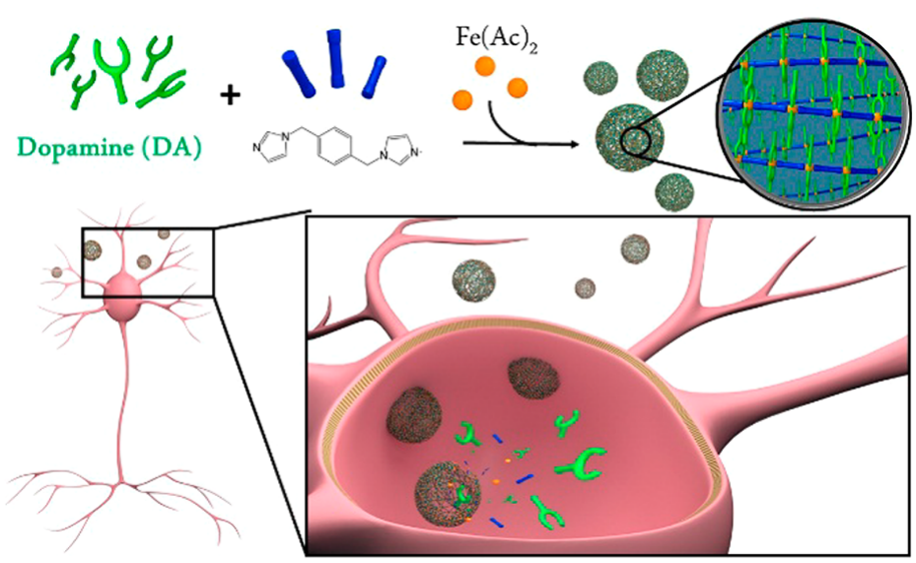

Dopamine (DA) is one of the main neurotransmitters found in the central nervous system and has a vital role in the function of dopaminergic (DArgic) neurons. A progressive loss of this specific subset of cells is one of the hallmarks of age-related neurodegenerative disorders such as Parkinson’s disease (PD). Symptomatic therapy for PD has been centered in the precursor l-DOPA administration, an amino acid precursor of DA that crosses the blood–brain barrier (BBB) while DA does not, although this approach presents medium- to long-term side effects. To overcome this limitation, DA-nanoencapsulation therapies are actively being searched as an alternative for DA replacement. However, overcoming the low yield of encapsulation and/or poor biodistribution/bioavailability of DA is still a current challenge. Herein, we report the synthesis of a family of neuromelanin bioinspired polymeric nanoparticles. Our system is based on the encapsulation of DA within nanoparticles through its reversible coordination complexation to iron metal nodes polymerized with a bis-imidazol ligand. Our methodology, in addition to being simple and inexpensive, results in DA loading efficiencies of up to 60%. *In vitro*, DA nanoscale coordination polymers (DA-NCPs) exhibited lower toxicity, degradation kinetics, and enhanced uptake by BE(2)-M17 DArgic cells compared to free DA. Direct infusion of the particles in the ventricle of rats *in vivo* showed a rapid distribution within the brain of healthy rats, leading to an increase in striatal DA levels. More importantly, after 4 days of nasal administrations with DA-NCPs equivalent to 200 μg of the free drug per day, the number and duration of apomorphine-induced rotations was significantly lower from that in either vehicle or DA-treated rats performed for comparison purposes. Overall, this study demonstrates the advantages of using nanostructured DA for DA-replacement therapy.

## Introduction

Parkinson’s disease (PD), the second most common neurodegenerative disorder in the world, affecting more than 10 million people,^1,2^ is characterized by motor symptoms including muscular rigidity, rest tremor, posture instability, and bradykinesia.^1^ The origin is associated with early prominent death of DArgic neurons typically of the substantia nigra pars compacta (SNpc),^3^ which leads to diminished levels of dopamine (3,4-dihydroxyphenethylamine, DA) in the striatum of these patients.^4−7^ Mainly due to restrictions for direct DA replacement, delivery of the precursor levodopa (l-3,4-dihydroxyphenylalanine, l-DOPA) has been used in the clinic for more than 50 years to mitigate Parkinson symptoms.^1^ However, long-term administration of this drug is associated with developing highly disabling complications such as drug-induced dyskinesia and motor fluctuations.^8−10^ Both complications are in part due to pulsatile l-DOPA administration and can be minimized with continuous and sustainable l-DOPA delivery by duodenal infusion.^11^ Evidence suggests that up to 50% of PD patients develop these complications after five years of chronic treatment with levodopa. Therefore, the development of alternative strategies to overcome these complications is an important challenge in modern medicine: among them, finding approaches for the delivery of DA through its nanoencapsulation.

Nanocarriers can help DA to avoid metabolism, cross the BBB, and promote a sustained release, reducing recurrent drug administration and, therefore, adverse effects. So far, different families of nanocarriers have been described with this aim, including chitosan^12^ and transferrin^13^ functionalized liposomes, (bio)hydrogels,^14,15^ chitosan nanoparticles,^16,17^ carbon nanotubes,^18^ alginate/magnetite hybrid beads,^19^ carbon dots,^20^ carboxymethyl cellulose coated magnetic nanoparticles,^21^ borneol and lactoferrin co-modified nanoparticles,^22^ and a polyvinylpyrrolidone-poly(acrylic acid) nanogel.^23^ The work by Gupta *etal*. deserves special mention, which reported DA-loaded poly lactic-*co*-glycolic acid (PLGA) nanoparticles able to reverse functional deficits in Parkinsonian rats.^24^ However, despite these advances, most of the nanocarriers used for DA replacement therapy often suffer from low yield of encapsulation and/or poor biodistribution and drug bioavailability, so alternative encapsulation approaches are required.

**Figure 1:**
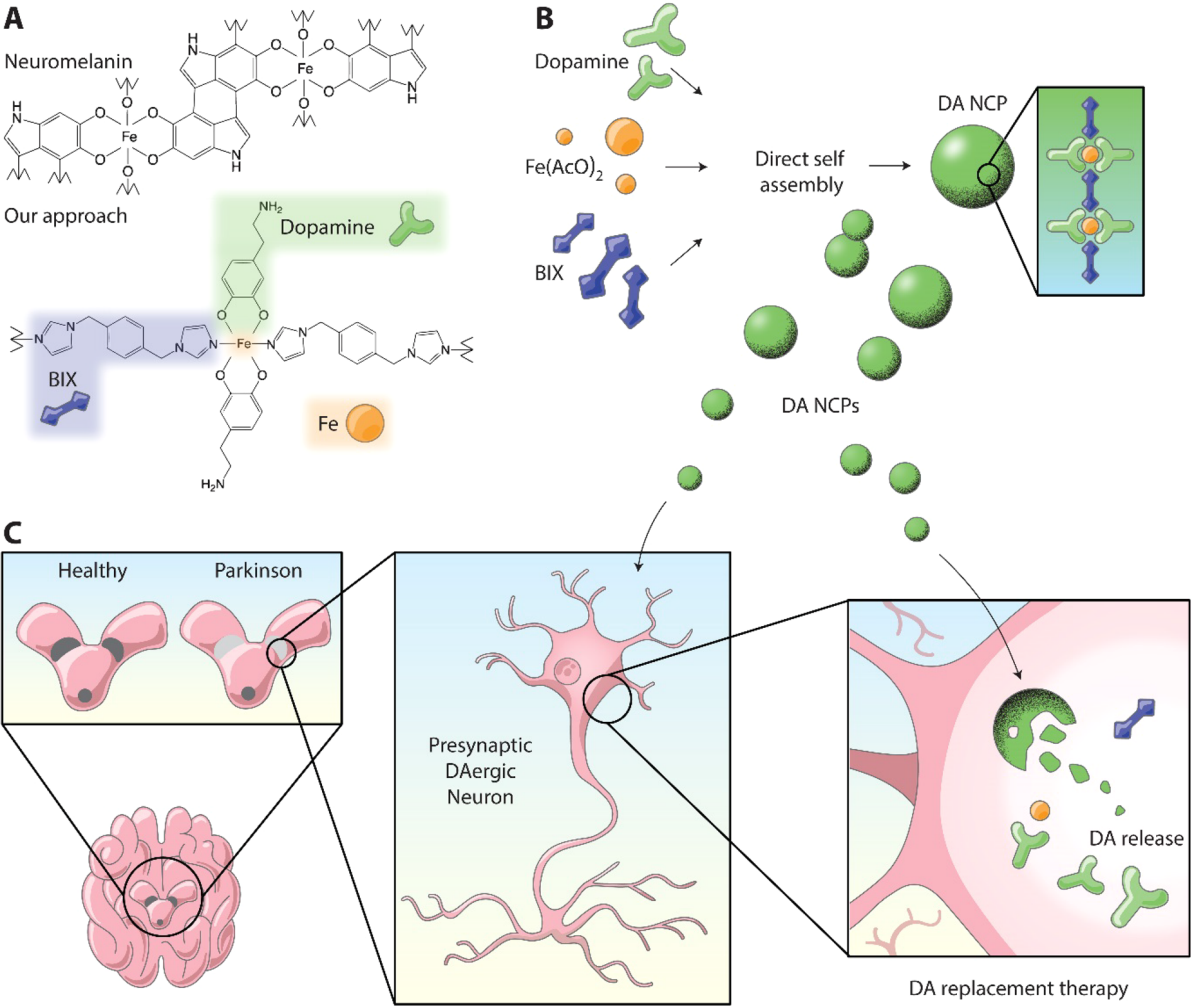
Bioinspired approach for dopamine (DA) replacement in Parkinson’s disease (PD). (a) Schematic composition of neuromelanin (NM) and the related nanoscale coordination polymer (DA-NCP) proposed herein. (b) Schematic representation of the synthesis of DA-NCPs from DA, Fe(AcO)_2_, and BIX. (c) Proposed model for DA-based replacement therapy in Parkinson’s disease using DA-NCPs.

Neuromelanin (NM) particles, found in DArgic neurons from the SNpc, incorporate large amounts of DA and iron ions, which allow for their use as a biomarker for PD. Therefore, we hypothesize that melanin granular pigments found in nature may offer inspiration to develop more effective DA nanocarriers.^25^ To mimic the synthesis of melanin-like nanoparticles in the lab, whose tentative formula is shown in Figure 1a,^26^ three main approaches have been so far reported. In the first approach, self-oxidation of DA under alkaline conditions results in the formation of colloidal polyDA (PDA) nanoparticles, which subsequently are doped with the appropriate metal salt (*postdoping approach*).^27−30^ Alternatively, one-pot polymerization of DA in the presence of transition-metal ions results in their inclusion in the final PDA (*in situ doping*).^31−33^ And the third approach involves the preformation of Fe(DA)_3_ monomeric complexes that systematically control the percentage of iron in the particles.^34−36^ Following these approaches, synthetic nanoparticles showing excellent biocompatibility and biodegradability have already been prepared and successfully used in phototherapy/drug delivery^37^ and imaging.^38−40^ However, none of them were found to be useful for the delivery of DA, as the polymerization and formation of the nanoparticles always take place through irreversible covalent bonds.

Herein we envision that this could be achieved with the formation of a tailor-made coordination polymer, where the central polymeric backbone is made of iron nodes linked to a bidentate ligand such as 1,4-bis(imidazol-1-ylmethyl)benzene (BIX) and DA is used as a counter ligand to complete the coordination sphere. The composition of the resulting nanoparticles (named from now on as DA-NCPs) is expected to have a structure inspired by NM, as shown in Figure 1a, but with the fundamental difference being that the incorporation of DA to the NP is reversible inside the cell. A schematic representation of the synthesis of the DA-NCPs nanoparticles and the expected biological function is shown in Figure 1b,c.

## Results and Discussion

### Synthesis and Characterization of DA-NCPs

**Figure 2:**
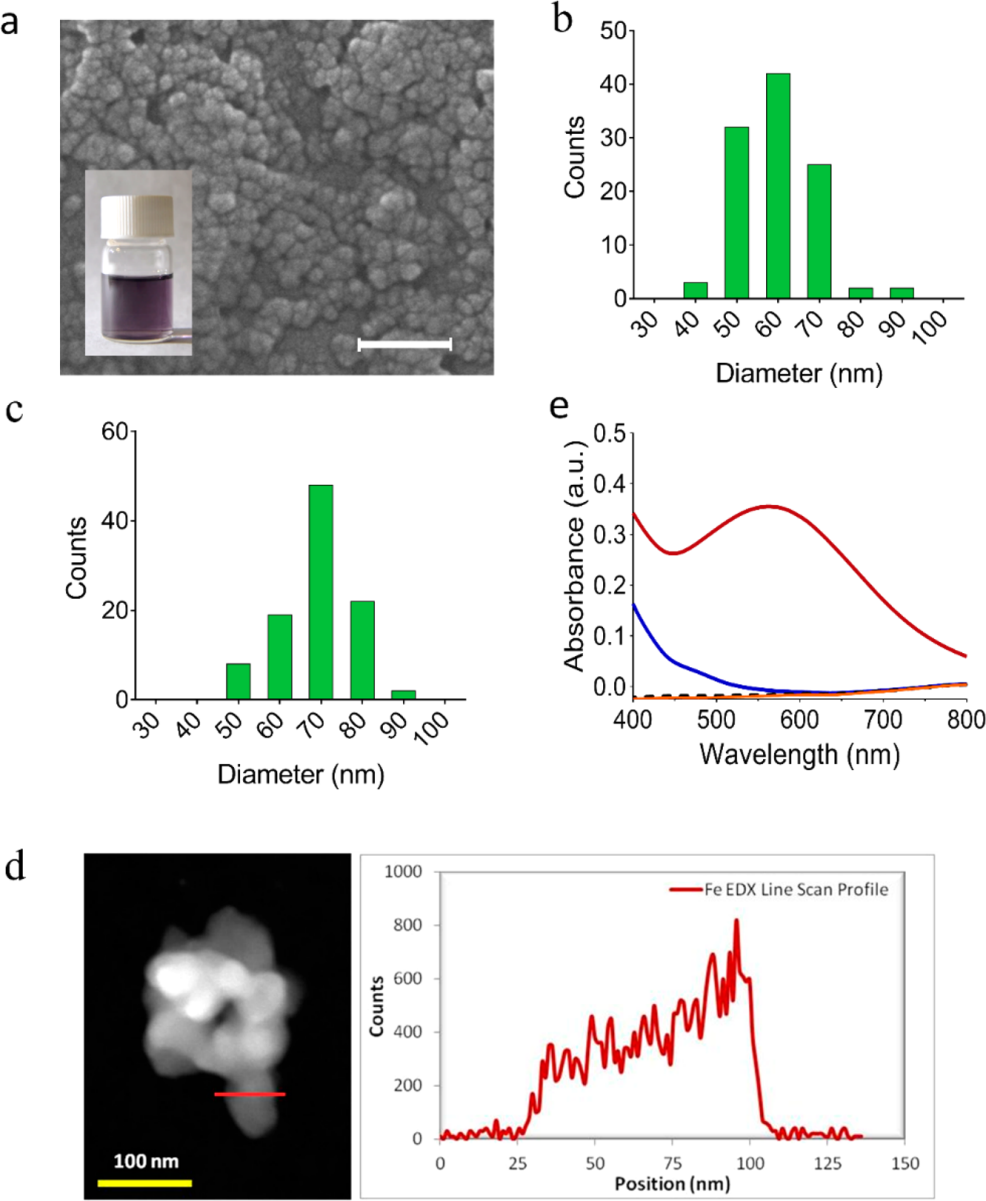
Chemical and morphological characterization of DA-NCPs. (a) SEM images of neuromelanin-inspired DA-NCPs. The inset shows a colloidal dispersion of the nanoparticles in ethanol. Scale bar, 200 nm. (b, c) Particle size distribution of DA-NCPs determined by (b) SEM and (c) negative-staining transmission electron microscopy (NS-TEM). (d) TEM image (left) and EDX profile of DA-NCPs along the dark red line marked (right). (e) UV–vis absorbance spectra of the particles (solid dark red line) and the corresponding chemical precursors: DA (black dashed line), BIX (solid blue line), and Fe(AcO)_2_ (solid orange line) determined at a concentration of 100 μg/mL in water.

DA-NCPs were prepared following a procedure already described for related catechol-based nanoparticles.^41,42^ In a typical synthesis, nanoparticles were obtained through a reaction of Fe(AcO)_2_ with DA hydrochloride in the presence of the ditopic ligand BIX. After 2 h under constant stirring at room temperature, a dark precipitate was isolated by centrifugation and washed gently with ethanol to eliminate all the unbound ligands (see details in Methods and Supporting Information). Scanning electron microscopy (SEM) and negative-staining transmission electron microscopy (NS-TEM) images of the obtained material revealed the presence of spherical nanoparticles with an average diameter of 64 ± 9.0 nm and 56 ± 9.0 nm, respectively (Figure 2a,c). These results were in agreement with dynamic light scattering (DLS) measurements of the DA-NCPs dispersed in ethanol, which showed a hydrodynamic diameter of 81 ± 4.0 nm (PDI: 0.124) (Supporting Information, Figure S1). Energy-dispersive X-ray analysis (EDX) profile of the TEM image clearly revealed the presence of iron homogeneously distributed along the whole nanoparticle (see Figure 2d), and Mössbauer spectroscopy studies confirmed that the iron was in the high-spin Fe(III) form (Supporting Information, Figure S2). This observation suggested that Fe(II) was oxidized to Fe(III) along with the nanoparticle synthesis. In agreement, the absorbance spectrum of DA-NCPs in the 400–800 nm range exhibited a characteristic maximum at 562 nm, which was attributed to the formation of Fe-bis-catechol coordination bonds (Figure 2e). This electronic modification has been attributed to a redox interplay between the metal ion and electroactive catechol ligands in air, as previously reported.^43^ The absence of diffraction peaks observed by powder X-ray diffraction (PXRD) indicated the amorphous nature of the nanoparticles (see Supporting Information, Figure S3).

The chemical characterization of DA-NCPs was addressed using different complementary techniques. Fourier transform infrared spectroscopy (FT-IR) confirmed the presence of DA and a BIX ligand coordinated to the Fe(III) metal ions (see Supporting Information, Figure S4). The FT-IR spectrum shows the disappearance of the bands corresponding to the catechol −OH groups of DA in the range of ν = 3000–3500 cm^–1^, reflecting their deprotonation, the stretching and bending vibrations of the amine group (3340 and 1618 cm^–1^, respectively), and the stretching vibrations of C–H (3040 cm^–1^) and C–C of the aromatic ring (1550 cm^–1^). Moreover, the vibration band assigned to a C–O stretch for the catechol coordinated to the metal appeared in the frequency range 1200–1300 cm^–1^. Additionally, typical vibrational bands of the BIX ligand (ν = 1520, 1262, 1105 cm^–1^) were observed in the FT-IR spectra. The ^1^H NMR spectrum of DA-NCPs dissolved in a deuterated methanol-HCl solution confirmed the presence of acetate counterions (see Supporting Information, Figure S5) and both ligands, although with a lower contribution of the BIX ligand than theoretically expected from the concentrations of the initial reactants. These results were quantitatively confirmed by high-performance liquid chromatography (HPLC) analysis of the product resulting upon treatment of DA-NCPs with an acidic solution; these analyses showed a content of 20% BIX (w/w) ligand. More interestingly, HPLC analysis using electrochemical detection (HPLC-ECD) confirmed the presence of 52.5 ± 7.2% DA in DA-NCPs nanoparticles (see Supporting Information and Materials and Methods section for experimental details). This exceptionally high value was confirmed with absorbance measurements using DA hydrochloride as a standard (Supporting Information, Figure S6). These analyses also revealed a very high loading efficiency (LE) of 69.7 ± 7.2%. For instance, this value is 6-fold higher compared with the LE of dopamine achieved with PLGA in previous reports. As far as iron is concerned, inductively coupled plasma-mass spectroscopy (ICP-MS) data confirmed a total amount of 10.2% in the nanoparticles. Finally, the combination of elemental analysis, ICP-MS, ^1^H NMR, and HPLC data yielded the following final tentative chemical formula for the nanoparticles: [Fe(DA)_1.6_(BIX)_0.5_(AcO)_0.2_(H_2_O)_1.9_] (see Supporting Information, Table S1). This formula differs from that theoretically expected on the basis of the initial reagents added (1 Fe:1 BIX:2 DA), which is attributed to their synthesis under out-of-equilibrium conditions induced by the fast precipitation process, a common phenomenon for this family of amorphous NCPs. Another plausible explanation for such differences could be the encapsulation of free ligand/solvent molecules within the particles present in the reaction solution, as already demonstrated in related systems.^44,45^ Such encapsulation ability has been confirmed by encapsulating, as a proof-of-concept, the recombinant epidermal growth factor (EGF) with an excellent encapsulation efficiency (EE) of 89.6%, and loading capacity (LC) of almost 7.7 μg of EGF per milligram of particles (Supporting Information, Materials and Methods and Figure S7).

### Colloidal Stability and DA Release Kinetics under Physiological Conditions

To simulate the colloidal stability of DA-NCPs in physiological environments, the nanoparticles were dispersed in water and phosphate-buffered saline (PBS) solution (with and without bovine serum albumin, BSA) at physiological conditions (0.5 mM).^46,47^ DLS measurements in water (Supporting Information, Table S2) showed higher hydrodynamic diameters (171 ± 4.2 nm; PDI: 0.208) than those obtained in ethanol (81 ± 4.0 nm; PDI: 0.124) or as a solid powder by SEM measurements (64 ± 9 nm). Moreover, even though the ξ-potential value in water was close to +25 mV, the colloidal solution was not stable and started to aggregate and precipitate over a relatively short period of time (sedimentation observed after 1 h). In PBS (20 mM PBS buffer at pH 7.4), the mean size values for nanoparticles indicated the presence of large aggregates (Supporting Information, Table S2) and showed a ξ-potential of −13.1 mV. The addition of 0.5 mM BSA, extensively used for dispersing inorganic and polymeric nanoparticles,^48,49^ successfully induced a disaggregation process and their stabilization without precipitation for several hours. The nanoparticles now showed a lower hydrodynamic diameter (103 ± 16.2 nm; PDI: 0.198), similar to that observed using culture medium containing fetal bovine serum (FBS) (Supporting Information, Table S2). The increase in size (24–28 nm) with respect to the value found in ethanol could be attributed to the formation of a protein corona around the nanoparticles.

We next investigated the release kinetics of DA from DA-NCPs nanoparticles after incubation for 24 h in a PBS–BSA buffer at 37 °C, using HPLC-ECD. At pH 7.4, a continuous release of DA, reminiscent of a standard saturation curve (Supporting Information, Figure S8), was observed with a cumulative release of 50% in the first 2 h (*t*_1/2_ = 2 h). Afterward, the release notably slowed down with an increase of 5% in the next 22 h. If the nanoparticles were dispersed at pH 5.5, the accumulative release of 50% was reached in less than 1 h. These results confirmed that the pH decrease induced a much faster release of the DA due to the lower stability of the coordinative bond between iron and catechol ligands. Finally, the release increased up to 85% in 10 h and 90% at 24 h. This pH dependence has been observed previously in mussel-inspired hydrogels with Fe-catechol complexes and in other Fe-containing polymeric nanoparticles.^30,50^

Worth mentioning is that the FBS used in the previous experiments was heat-inactivated and does not fully represent the real physiological conditions found in the blood and plasma under *in vivo* conditions. For this reason, we also performed an additional experiment to study the colloidal stability of the DA-NCPs in the presence of nontreated human plasma. For this, an aliquot of DA-NCPs was suspended in 1 mL of ethanol or in 1 mL of nontreated human plasma. Afterward, the hydrodynamic diameter of the nanoparticles was evaluated at different time points of incubation at 37 °C. No relevant changes in the size distribution of the nanoparticles up to 6 h incubation under these conditions were detected. Only a small shift toward higher dimensions was observed, compatible with the progressive formation of the protein corona due to the high concentration of proteins in the human plasma. As expected, the presence of these proteins was visible as a secondary peak (peak 2, in Figure S9) with a size of ∼40 nm.

### Evaluation of the Cytotoxicity of DA-NCPs against DArgic Neurons

To evaluate the effect of DA-NCPs on DArgic cell survival, *in vitro* cytotoxicity assays with (BE)2-M17 cells were performed. (BE)2-M17 is a neuroblastoma-derived DArgic cell line that expresses large amounts of key proteins essential for the DArgic metabolism and, therefore, extensively used as a cellular model to study DA metabolism.^51^ Treatment of the (BE)2-M17 cells with DA-NCPs did not lead to any appreciable cell viability reduction after 48 h of incubation at concentrations up to 100 μg/mL (expressed as DA concentration) (see Supporting Information, Figure S10). The same concentrations of free DA in the range from 0.01 to 100 μg/mL showed comparable results. However, slightly higher levels of the free neurotransmitter (*i*.*e*., 200 and 250 μg/mL) caused a dramatic death of DArgic cells with more than 85% reduction in the cell viability (Supporting Information, Figure S10). Interestingly, comparable concentrations of DA-NCPs caused lower cytotoxic effects. The toxicity found for the highest concentrations of DA was attributed to the intracellular metabolism of DA (conversion of DA to 3,4-dihydroxyphenylacetaldehyde, DOPAL) that generates hydrogen peroxide and, therefore, reactive oxygen species (ROS) effects. This process can take place both enzymatically (monoamine oxidase, MAO) or nonenzymatically. ROS can also be generated upon DA oxidation; when free DA cytosol levels exceed a certain threshold level, DA can spontaneously autoxidize, producing *o*-quinone molecules.^52^ These species are very reactive and may contribute to increasing the levels of oxidative stress inside the cells. To confirm this fact, intracellular ROS formation in BE(2)-M17 cells was also measured after treatment with DA or DA-NCPs, through the 2′,7′-dichlorofluorescein diacetate (DCFDA) assay (see Materials and Methods).^53^ After a 24 h treatment, the 300-fold DCF fluorescence increase found for the highest free DA concentration (see Supporting Information, Figure S11) confirmed the ROS production.^24^ Ascorbic acid (AA), at physiological concentrations of 20 μg/mL, was required to drastically reduce ROS effects of H_2_O_2_. Interestingly, DA-NCPs showed a lower DCF fluorescence increase (200-fold) even considering the possible oxidative stress coming from iron ions (Supporting Information, Figure S12).

On the basis of these findings, we can state that our nanoparticles did not show a reduction in cell viability at the assayed DA concentrations in the range 0.01 to 10 μg/mL^54^ and that DA nanostructuring into DA-NCPs reduced the toxicity of the monoamine against DArgic cells. Similar observations were reported by Pahuja *etal*.,^24^ who demonstrated a reduction in the toxicity and oxidative damage induction of l-DOPA upon encapsulation into PLGA nanoparticles. Evaluation of the toxicity of the iron salt and the linker ligand was performed in (BE)2-M17 cell culture using concentrations ranging from 0 to 100 μg/mL for comparison purposes (see Supporting Information, Figure S13), with no cytotoxicity detected even at the highest concentration assayed (100 μg/mL). Finally, and for comparison purposes, we also studied the cytotoxicity of DA and DA-NCPs in a non-DArgic cell line such as HeLa cells (Supporting Information, Figure S13), where as expected, IC_50_ was smaller for both cases (see Supporting Information, Table S3).

### Uptake of DA-NCPs by DArgic (BE)2-M17 Cells

The DA-NCPs’ uptake kinetics by DArgic (BE)2-M17 cells was studied and compared to free DA. To perform the experiments, cells were exposed to increasing concentrations of DA or DA-NCPs and incubated for 2, 6, and 24 h. Immediately after incubation, cells were collected and the intracellular concentration of DA was measured by HPLC-ECD (for more details, see Supporting Information and Materials and Methods).

#### Free DA

**Figure 3:**
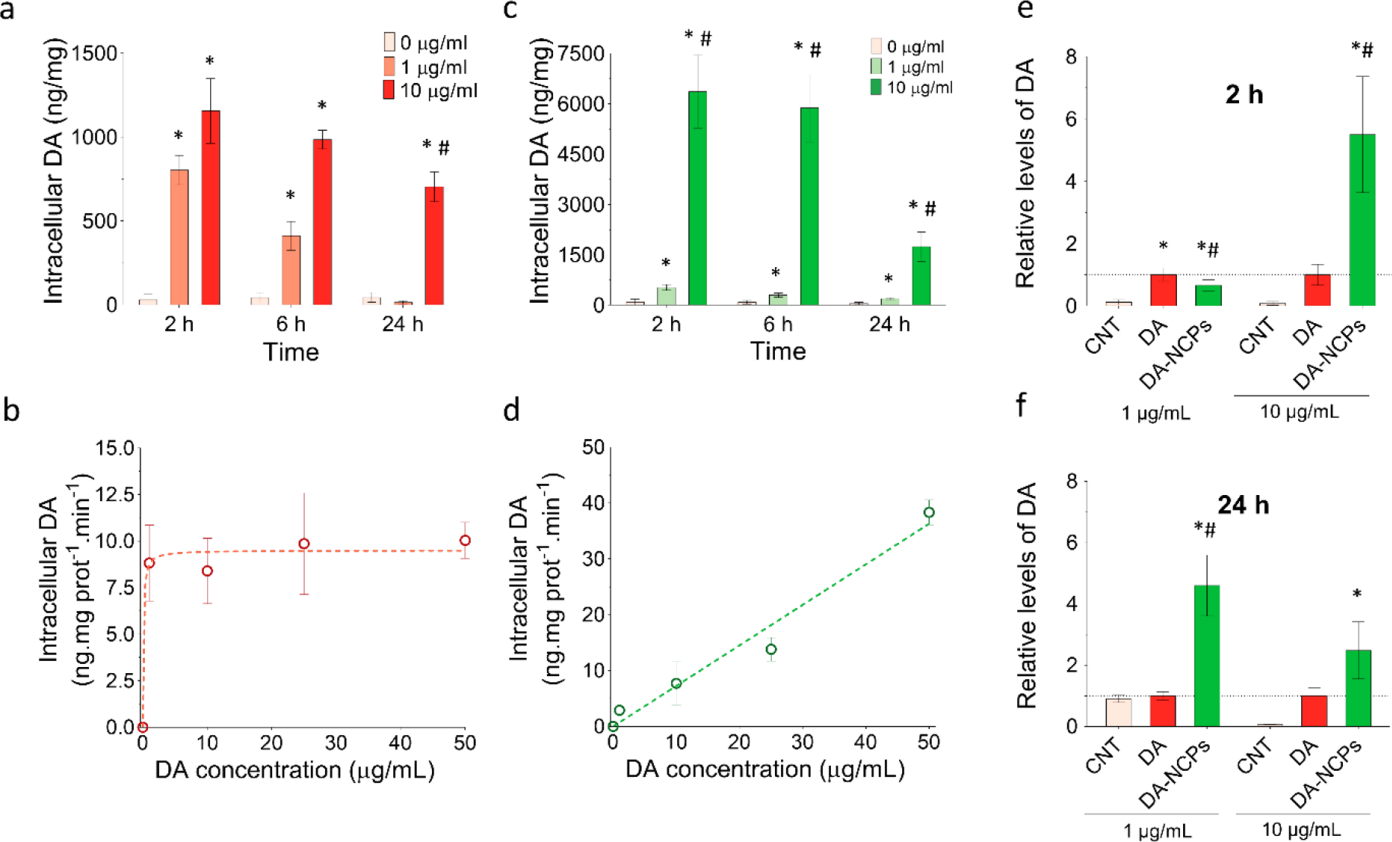
Uptake kinetics of DA and DA-NCPs by DArgic (BE)2-M17 cells. (a) Intracellular DA levels determined by HPLC-ECD after incubation of (BE)2-M17 cells for 2, 6, and 24 h in the absence (0 μg/mL, shown in light pink) or in the presence of DA at two different concentrations (1 and 10 μg/mL of DA, shown in light red and red, respectively). (b) Rate of DA uptake after the treatment of the cells with DA at 1, 10, 25, and 50 μg/mL for 2 h. (c) Intracellular DA levels after incubation of (BE)2-M17 cells for 2, 6, and 24 h in the absence (0 μg/mL, shown in light pink) or in the presence of nanostructured DA (DA-NCPs) at two different concentrations (concentration equivalent to 1 and 10 μg/mL of DA, shown in light green and green, respectively). (d) Rate of DA uptake after the treatment of the cells with DA-NCPs at concentrations equivalent to DA concentrations of 1, 10, 25, and 50 μg/mL for 2 h. (e, f) Relative intracellular levels of DA after incubation for (e) 2 h and (f) 24 h in the absence (0 μg/mL, shown in light pink) or in the presence of DA or DA-NCPs at two different concentrations (concentration equivalent to 1 and 10 μg/mL). In all cases, values are mean ± SEM. In (a) and (c), *p < 0.05, compared to 0 μg/mL; ^#^*p* < 0.05, compared to treatment with 1 μg/mL; a three-way ANOVA (full factorial) and Sidak correction was applied to the log-transformed data. In (e) and (f), *p < 0.05, compared to control; ^#^*p* < 0.05, compared to DA (one-way ANOVA and Turkey’s *posthoc* test).

Incubation of (BE)2-M17 cells in the presence of 1 μg/mL of free DA resulted in a rapid concentration-dependent increase in intracellular DA level after 2 h of incubation (Figure 3a). This fact could be attributed to a large amount of DA transporters (DATs) that DArgic neurons use to control extracellular DA levels by pumping the extracellular DA from the synaptic cleft to the cytosol.^55^ As shown in Figure 3a, at longer incubations times (6 and 24 h), a progressive decrease in the total intracellular DA concentration was observed. This decrease was accompanied by a constant reduction in the extracellular DA levels (see Supporting Information, Figure S14). The rate of uptake of DA through DAT is assumed to follow characteristic saturation kinetics.^56^ To confirm this, the experimental data obtained for the different DA concentrations used at 2 h were plotted and precisely fitted to Michaelis–Menten kinetics, with approximate *K*_m_ and *V*_max_ values of 0.1 μg/mL and 10.0 ng·mg prot^–1^·min^–1^, respectively (Figure 3b). All in all, the results suggested that DA was actively taken up and metabolized by the DArgic (BE)2-M17 cells due to the action of the intracellular DArgic mechanism.

#### DA-NCPs

Incubation of cells with DA-NCPs accounting overall for the same concentration of DA showed different intracellular profiles (Figure 3c,d). At 2 h and the lowest concentration (1 μg/mL), the DA internalized amount was slightly lower than that of free DA. Incubation of cells with higher doses (10 μg/mL) for the same period of time induced an intracellular level of DA up to 6360 ng/mg. This concentration was 6-fold higher than that obtained for free DA (1156 ng/mg) under the same concentration and experimental conditions. Moreover, the plot of DA internalization *vs* the initial external concentration used showed a nonsaturable dose–response mechanism instead of the characteristic Michaelis–Menten kinetics (Figure 3d), suggesting the presence of another pathway for nanoparticle uptake. Similar results and differences were found at 6 h of incubation, in agreement with the presence of DA outside the cells at this incubation time (Figure S14).

Note that the total intracellular DA determined by HPLC-ECD represents the sum of vesicular DA and the fraction of DA present in the cytoplasm. In fact, only the excess of DA accumulated in the cytoplasm of the cells is available and can be degraded by the MAO. Therefore, the overall effect is a progressive and slow decline of intracellular DA levels that do not fully correspond to the extracellular DA concentration. Finally, after 24 h of incubation in the presence of 1 μg/mL, DA-NCPs-exposed cells displayed a 4-fold amount of internalization compared to control and free DA-treated cells (Figure 3a and f), where depletion of the extracellular DA caused normalization of the intracellular levels of the neurotransmitter. This finding is of particular significance as demonstrated by the enhanced intracellular retention of the neurotransmitter for longer periods of time upon nanostructuring.

### Metabolism Kinetics of DA and DA-NCPs by DArgic Cells

**Figure 4:**
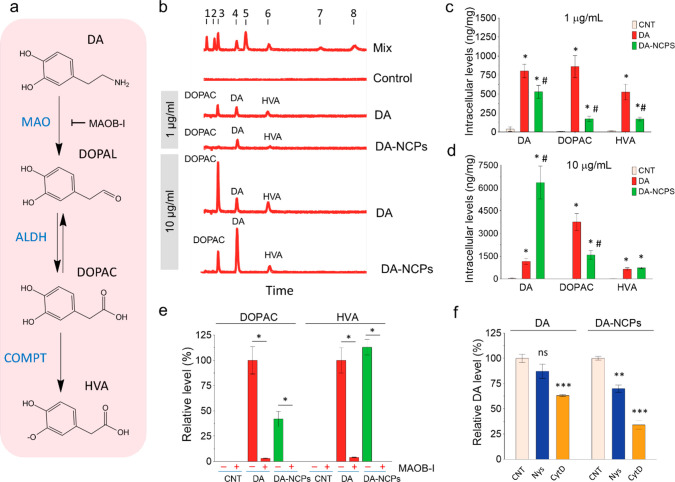
Metabolism kinetics of DA and DA-NCPs in DArgic neurons. (a) Principal degradation pathways of DA in DArgic neurons and main products obtained. (b) Representative HPLC-ECD chromatograms showing those DA derivatives found in (BE)2-M17 cells after treatment with two different concentrations (1 and 10 μg/mL) of free DA or DA-NCPs for 2 h. The mix of standards (mix) contained 1: l-DOPA; 2: DHBA, 3: DOPAC; 4: DA; 5: 5HIIA; 6: HVA, 7: 3-MT, and 8: 5HT. (c, d) Intracellular levels of DA and its metabolites (DOPAC and HVA) after 2 h of incubation with (c) 1 μg/mL or (d) 10 μg/mL of free DA or DA-NCPs. (e) Effect of deprenyl R, a specific monoamine oxidase inhibitor (MAOB-I), on DA metabolism. (f) Effects of chemical inhibition for selected endocytic pathways on DA uptake. Nys: nystatin; CytD: cytochalasin D. In all cases, values are mean ± SEM and *n* = 6 independent experiments, unless otherwise specified. In (c) and (d), *p < 0.05, compared to CNT; ^#^*p* < 0.05, compared to treatment with DA (three-way ANOVA (full factorial) and Sidak correction was applied to the log-transformed data). In (e), *p < 0.0001, when compared between conditions with and without MAOB-I (two-tailed *t*-test). In (f), **p < 0.01, ***p < 0.001, ns (nonsignificant), when compared to CNT (two-tailed *t*-test); *n* = 3 independent experiments.

The death of DArgic neurons in the substantia nigra leads to deficient striatal DA levels responsible for the cardinal motor symptoms of PD (*i*.*e*., including bradykinesia, tremor, and rigidity).^57^ Under normal conditions, the stimulation of healthy DArgic neurons causes the release of the synaptic vesicles into the synaptic cleft, where the neurotransmitter interacts with the postsynaptic DA receptors or regulatory presynaptic DA autoreceptors.^58−62^ When DA reaches the cytoplasm of DArgic neurons, this neurotransmitter is rapidly stored back into the synaptic DA storage vesicles. However, an excess of DA in the cytoplasm of the cell is metabolized into 3,4-dihydroxyphenylacetic acid (DOPAC) by monoamine oxidase B enzyme (MAO-B)^58^ and aldehyde dehydrogenase (ALDH). Then DOPAC is converted into homovanillic acid (HVA) by the action of catechol-O-methyltransferase (COMT) (see Figure 4a).^59^ Besides, synaptic cleft DA can be taken up by surrounding glial cells and be degraded by MAO and also by COMT, which transforms DOPAC into HVA, one of the main degradation products of DA (Figure 4a).

To study this metabolism and degradation kinetics, the intracellular concentration of DA and derived metabolites was determined using HPLC-ECD in (BE)2-M17 cells incubated with free DA and DA-NCPs for 2 h and at two concentrations (1 and 10 μg/mL). As shown in Figure 4b, in both cases levels of DA and metabolites increased though DOPAC and HVA levels were 5.3 times and 3.1 times, respectively, lower for DA-NCPs than for free DA (Figure 4b,c). In other words, at 1 μg/mL the DA/(DOPAC+HVA) ratios for free DA or DA-NCPs were 0.58 and 1.54, respectively (∼2.6-fold reduction for DA-NCPs). This difference was accentuated at 10 μg/mL (Figure 4d), where the DA/(DOPAC+HVA) ratios were 0.30 and 2.80 for DA and DA-NCPs, respectively (9.3-fold increase).

As anticipated, free DA exhibited a progressive reduction in DOPAC and HVA levels with time for the 1 μg/mL concentration (see Supporting Information, Figure S15), while a higher DOPAC production and less abrupt reduction were observed at 10 μg/mL (HVA levels remained quite homogeneous with time). A similar tendency was found for DA-NCPs at the lowest concentration, though at higher concentrations (10 μg/mL) the generation of metabolites remained constant and only fell after 24 h. These observations could be indicative of the DA-reservoir properties of DA-NCPs and a relatively slow release of free DA from the nanoformulation, which limits the formation of metabolites. Another essential factor to determine was how DA-NCPs (or DA released from them) could be metabolized by MAO-B. For this, we incubated (BE)2-M17 cells with the DA-NCPs in the presence of 2 μM deprenyl-R, a specific monoamine oxidase B inhibitor (MAOB-I). As shown in Figure 4e, the levels of DOPAC and HVA fell following treatment with this particular chemical inhibitor. Similar results were observed in the case of free DA-treated cells, suggesting that DA released from the nanoparticles could be actively processed by the oxidative DArgic mechanism.

Finally, we evaluated the uptake of DA or DA-NCPs in the presence of specific inhibitors of endocytic pathways. As shown in Figure 4f, the presence of cytochalasin D (CytD) induced a small decrease in DA uptake for free DA, while both nystatin (Nys) and CytD caused an important reduction in the uptake of DA-NCPs. These results indicated that an essential fraction of the internalization of DA-NCPs was mediated by specific endocytic pathways. Moreover, actin/dynamin interactions play a central role in the recycling of DAT *via* the bulk endocytic route. In fact, treatment of adult dopaminergic neurons with CytD (used to disrupt the actin filaments) significantly reduces DAT surface levels to 69.9 ± 5.1% of control levels.^63^ This reduction in the number of receptors on the surface of the cells is compatible with the decrease in the DA uptake (63.1 ± 2.8% compared to control) observed in our experiments upon CytD treatment.

### *In Vivo* Biodistribution and Metabolism of DA-NCPs within the Brain

**Figure 5:**
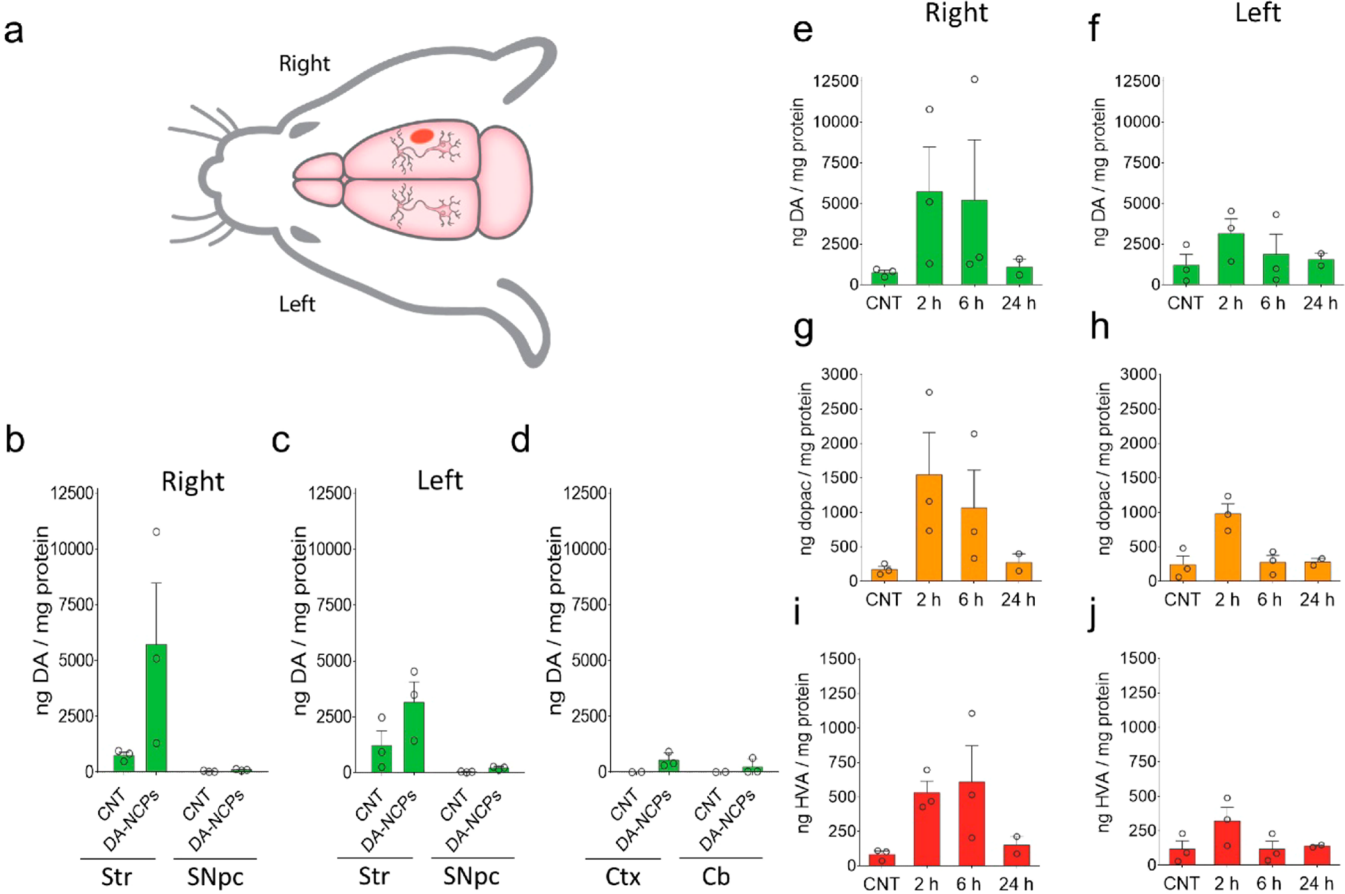
Biodistribution and metabolism of DA-NCPs in the brain after i.c.v. injection. (a) Schematic representation of the rat brain depicting the DA-NCPs injection site (red dot) and the location of the different regions analyzed (*vide infra*). (b, c) DA levels in the Str and SNpc (b) ipsilateral (right) or (c) contralateral (left) to the injected side after 2 h post-DA-NCPs administration. (d) DA levels in the Ctx and Cb after 2 h post-DA-NCPs injection. (e–j) Time-dependent metabolism of DA-NCPs after the i.c.v. injection of the nanoparticles. (e and f) DA, (g and h) dihydroxyphenylacetic acid (DOPAC), and (i and j) homovanillic acid (HVA) levels determined in the right and left striatum at different time points post-DA-NCPs injection. Data represent mean ± SEM. In all cases *n* = 3, unless otherwise indicated.

To examine the *in vivo* brain biodistribution of DA-NCPs, a single dose was infused in the lateral ventricle of healthy adult male Sprague–Dawley rats (Figure 5a) through a surgical cannula installed in the right ventricle. The i.c.v. infusion of DA and DA agonist has been attempted in the past in rodents, primate models, and human patients of PD as an alternative to conventional treatments.^64^ Venna and co-workers reported a good tolerance to continuous i.c.v. DA infusion in patients treated over 1 year.^65^ This continuous DA delivery resulted in a more stable treatment effect without the “on–off” fluctuations of traditional oral therapy.

The materials were well tolerated by the animals, as no adverse effects nor increased mortality was observed during nanoparticle administration, in agreement with the low *in vitro* toxicity values and the absence of systemic toxicity previously reported. The animals were then euthanized to measure the DA and DA metabolite levels of different brain regions using HPLC-ECD at different time points after intracerebroventricular (i.c.v.) injection. As shown in Figure 5, distribution of DA-NCPs from the right lateral ventricle to the surrounding neuronal tissues occurred mostly within the first 2 h, with a notable increase in DA levels observed in the striatum and SNpc (Figure 5b,c and Figure S16). This observation suggested that DA released from the nanoparticles was readily bioavailable and could be efficiently incorporated by nigrostriatal DArgic neurons *in vivo*.

It is worth mentioning that higher amounts of DA could be detected in the right striatum ipsilateral to the site of administration, in comparison with the levels detected for the left striatum. In addition to the nigrostriatal track, we identified small amounts of DA in other regions of the brain, such as the cortex or the cerebellum (Figure 5d). Striatal DA concentration recovered its basal levels after 24 h postinjection, indicating that DA-NCPs were completely cleared and/or metabolized in the brain by DArgic neurons or by surrounding glial cells (Figure 5e,f). Thus, we further investigated the DA-NCPs metabolism in the brain by measuring the levels of the two main metabolites, DOPAC and HVA. As shown in Figure 5g–j, DOPAC and HVA levels showed an increase in the right striatum after 2 and 6 h postadministration, in agreement with the *in vitro* metabolism experiments using DArgic cells.

### DA-NCPs Display Magnetic Resonance Imaging Properties

**Figure 6:**
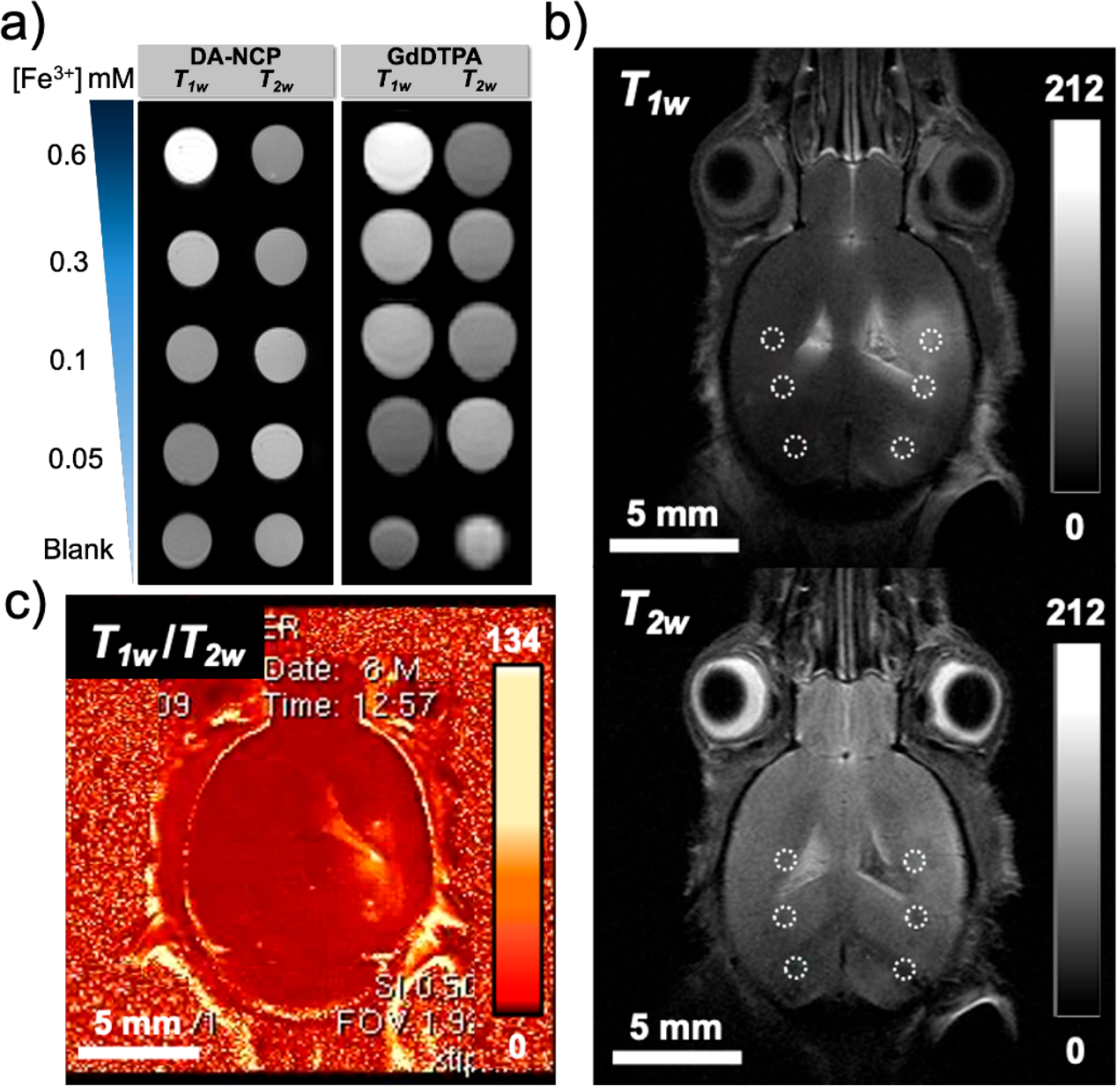
Magnetic resonance imaging (MRI) experiments. (a) *In vitro T*_1w_ and *T*_2w_ MRI of phantoms with DA-NCPs in comparison with commercial GdDTPA at decreasing metal concentration. (b) *Ex vivo* DA-NCPs *T*_1w_ (top) and *T*_2w_ (bottom) MRI obtained after stereotactic injection of DA-NCPs. (c) Postprocessing of *T*_1w_ and *T*_2w_ images by an algebraic algorithm of imaging division (*T*_1w_/*T*_2w_). For the *ex vivo* experiments, DA-NCPs and GdDTPA were stereotactically injected in brain parenchyma. The dashed circles indicate the selection of ROIs for the RCE calculation. Color scales show the range of pixel values.

Longitudinal (*r*_1_) and transversal (*r*_2_) relaxivity values were determined at 7 T by measuring corresponding relaxation rates in phantoms containing different concentrations of DA-NCPs dispersed in PBS/agarose 1% solution at pH 7.4 (*T*_1_ and *T*_2_ map sequences, see detailed protocols in the Supporting Information; representations of *R*_1_, *R*_2_*vs* metal concentration are shown in Supporting Information, Figure S17). As presented in Figure 6a, samples showed increased *T*_1w_ enhancement (brighter signal) with the increase of the metal ion concentration. The *r*_1_ value was determined to be 5.1 ± 0.6 mM^–1^ s^–1^ for DA-NCPs, which was comparable with related iron-based NCPs (*i*.*e*., *r*_1_ = 4.4 mM^–1^ s^–1^ for Fe-NCPs),^66,67^ previously reported iron-based coordination polymers,^68,69^ paramagnetic molecular complexes (3.0 mM^–1^ s^–1^),^70^ or even commercial gadolinium diethylenetriaminepentacetate (GdDTPA) contrast agents (CAs) (Magnevist, 4.4 ± 0.2 mM^–1^ s^–1^) (Figure 6b). Such an exceptional response could be directly related to the ability of catechol-based ligands to maximize second-sphere interactions with water molecules and therefore enhance the *T*_1w_ MRI signal through hydrogen bonding with the oxygen atoms of the Fe–O–R linkages.^71^ Moreover, DA-NCPs probably contained water molecules coordinated to the metal sites, as well as free water molecules, which probably exchange with surrounding water molecules, diffusing through the polymeric matrix.^72^ Encouraged by these exceptional results and considering that phantom results are not always fully comparable with contrast enhancement in real tissues,^73−75^*ex vivo* studies with DA-NCPs were conducted following a methodology previously reported.^76^ For this, PBS–BSA dispersions of DA-NCPs and commercial GdDTPA (for comparison purposes) were intracranially administered *post mortem* to euthanized mice. *T*_1w_ and *T*_2w_ MRI was subsequently acquired, and relative contrast enhancement (RCE) calculated (see Table S4) (details about calculations and protocol can be found in the Supporting Information and Materials and Methods section).

Three different regions of interest (ROIs) were selected in *T*_1w_ and *T*_2w_ images, corresponding to the CA administration points (Figure 6b). DA-NCPs presented higher RCE *T*_1_ in comparison with the commercial CA GdDTPA and comparable RCE *T*_2_. The postprocessing of *T*_1w_ and *T*_2w_ images by an algebraic algorithm of imaging division (*T*_1w_/*T*_2w_) showed a clear brightness area when DA-NCPs were injected (Figure 6c) into the brain, confirming the excellent behavior of DA-NCPs for MRI monitoring.

### Intranasal Administration of DA-NCPs

**Figure 7:**
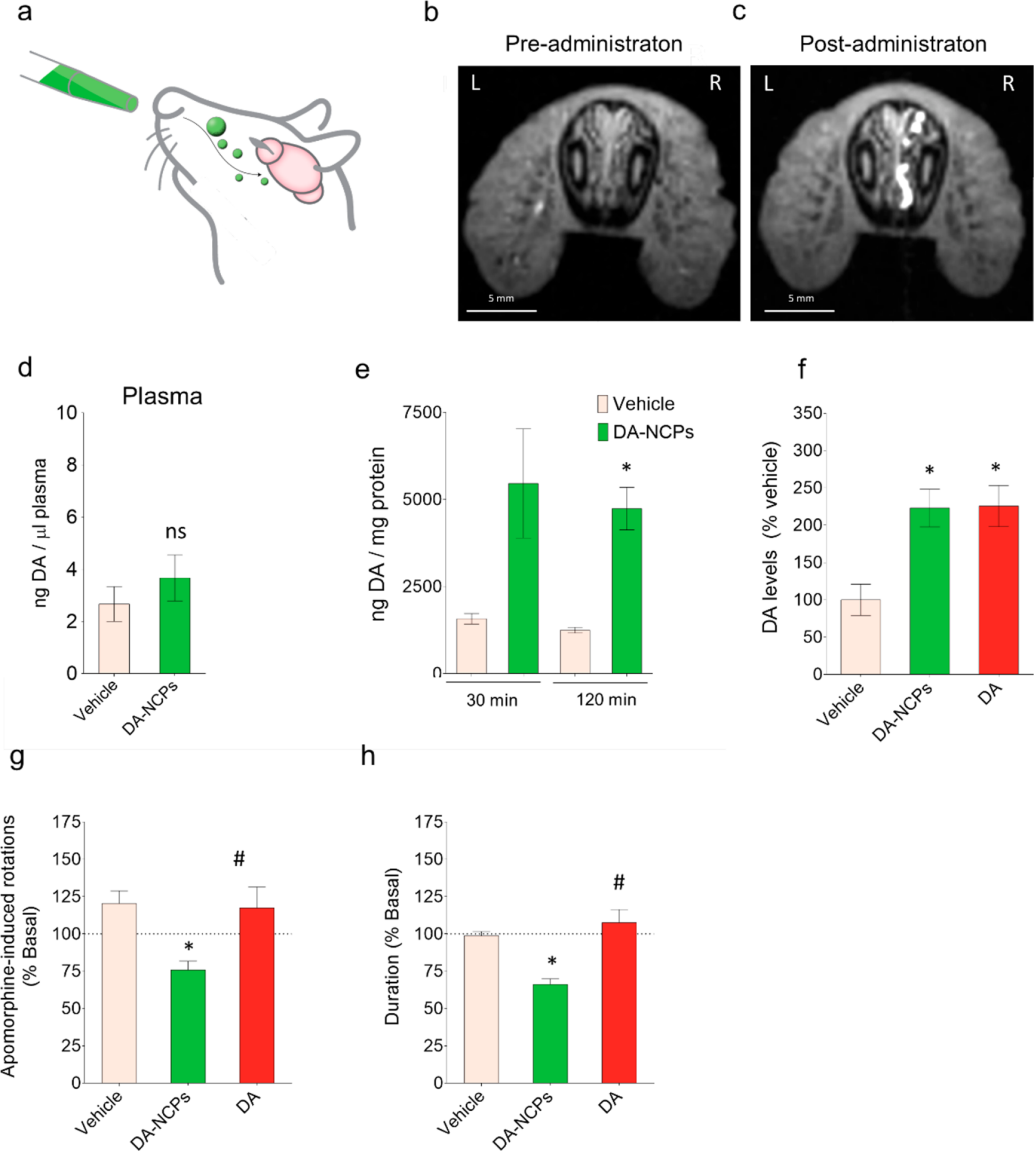
Intranasal administration of DA-NCPs and pharmacological effects on the 6-OHDA rat model of PD. (a) Schematic representation for the intranasal administration of the DA-NCPs. (b–f) Biodistribution of intranasally applied DA-NCPs. *T*_1w_ MRI from the nasal cavity of a rat obtained (b) before (pretreatment) and (c) after (post-treatment) intranasal application of the nanoparticles. (d) DA levels detected in the plasma of healthy rats treated with vehicle or with a dose of DA-NCPs equivalent to 50 μg of free DA. (e) DA concentrations in the right striatum of healthy rats determined at two different time points (30 min and 2 h) post-DA-NCP treatment. Values are expressed as ng of DA/mg of protein. Data are presented as mean ± SEM. (f) Levels of DA in the right striatum of healthy rats. The test groups include animals treated with vehicle or intranasally administered with a dose of DA or DA-NCPs (dose equivalent to 50 μg of DA). Values are presented as percentage of DA compared to the vehicle-treated animals. Data are presented as mean ± SEM. (g) Apomorphine-induced rotational behavior of 6-OHDA-lesioned rats. Data are percentage of apomorphine-induced rotations determined 24 h postadministration. The net number of contralateral rotations was calculated as total left 360° – total right 360° turns, and data are presented as percentage of rotations in comparison with values achieved in basal conditions before treatment. Duration of the apomorphine-induced rotational behavior. Data are expressed as percentage of the time achieved in the basal experiment before treatment. The apomorphine-induced rotation test was performed in 6-OHDA-lesioned rats previously treated with repeated administrations of vehicle (*n* = 6), DA (*n* = 6), or DA-NCPs (*n* = 6). During the semichronic treatment, each animal received a total dose of DA or DA-NCPs equivalent to 800 μg of free DA, administered on 4 consecutive days (200 μg/day). In all cases, values are shown as mean ± SEM. In (d) and (e) ns (nonsignificant) when compared to vehicle; *p < 0.05, compared to vehicle (two-tailed *t*-test). In (f)–(h), ns, *p < 0.05, compared to vehicle; ^#^*p* < 0.05, compared to treatment with DA-NCPs (one-way ANOVA and Tukey’s *posthoc* test).

The direct infusion of DA-NCPs demonstrated rapid biodistribution and pharmacokinetic potential. However, this administration route is not an appropriate choice for the continued treatment of PD patients since it is invasive and requires a surgical procedure for the insertion of the i.c.v. device. So, the potential application of DA-NCPs for nose-to-brain delivery of DA was then evaluated (Figure 7a) in rats. First, we aimed to track the biological pathway followed by the nanoparticles after their intranasal administration using *in vivo* MRI. As shown in Figure 7a and b, a strong *T*_1w_ signal was clearly observed in the right nasal cavity of the rat, near the respiratory epithelia. *T*_1_ values measured within ROIs from three consecutive sections were significantly decreased after different preadministration of DA-NCPs (*T*_1_ = 707 ± 119 ms) compared to postadministration values (1631 ± 91 ms, *t*-test: *p* = 0.003). Previous intranasal studies demonstrate that drugs gain direct access to the central nervous system (CNS) *via* direct pathways from the nasal cavity.^77^ A more detailed analysis of different longitudinal and coronal sections of the anterior rat head and nasal cavities (Figure S18a,b) elucidated the presence of a strong DA-NCPs signal in the right duct of the vomeronasal organ. These results clearly showed that DA-NCPs were in contact with the mucosa and the epithelia of the nasal cavity immediately before they were transferred into the CNS *via* the olfactory bulb.^78^

Plasma DA levels of healthy rats after 2 h of administration did not show relevant differences between samples taken from rats treated with DA-NCPs (dose equivalent to 50 μg of free DA) or only with the vehicle (Figure 7d), indicating that DA was not transferred to systemic circulation after this time. By contrast, significantly higher amounts of striatal DA were detected in the ipsilateral side of the rat brains after 30 min and 2 h of unilateral DA-NCPs nasal administration (Figure 7e). The maximum DA concentration was achieved after 30 min with a 3.5-fold increase in comparison with basal striatal DA levels, consistent with observations noted in earlier intranasal investigations.^78^ Time evolution analysis of HVA and DOPAC metabolites in the same samples nicely correlated with that of DA, showing a clear increase of these two metabolites associated with the simultaneous activation of the DA catabolism in the right striatum. Interestingly, no significant increase in DA was observed in the contralateral striatum after unilateral administration of DA-NCPs (Figure S18c). Similar to DA-NCPs, unilateral intranasal administration of an equivalent dose of free DA into the right nostril also led to a clear increase of the striatal DA, reaching comparable levels to those observed in the case of the nanoparticles (Figure 7f).

### Pharmacological Effect in 6-HydroxyDA (6-OHDA)-Lesioned Rats

To determine if these increased levels of DA in the striatum of DA-NCPs-treated rats are physiologically relevant, we examined the pharmacological effect of the nanoparticles in 6-hydroxyDA (6-OHDA)-lesioned rats, a PD model characterized by a typical unilateral drug-induced degeneration of the nigrostriatal DArgic neurons on the same side as neurotoxin injection. Measurements following intraperitoneal injection of apomorphine, a potent DArgic agonist, in the 6-OHDA-lesioned rats revealed a characteristic contralateral turning behavior (average of rotations in all the groups analyzed in the basal test: 249 ± 146 contralateral turns; average duration of the rotational behavior: 57 ± 11 min) in response to the stimulation of the DA receptors. After 4 days of nasal administrations with DA-NCPs equivalent to 200 μg of the free drug per day, the number and duration of apomorphine-induced rotations were significantly lower from that in either vehicle- or DA-treated rats performed for comparison purposes (Figure 7g,h). This shortening in the motor response is compatible with the continuous presence of DA in the nigrostriatal pathway, indicating desensitization in unilateral 6-OHDA-lesioned rats continuously treated with the nanoparticles. Compared to DA-treated animals, the different therapeutic response observed in DA-NCPs might be attributed to their more sustained release potential.

## Conclusions

Inspired by natural NM, we have reported a family of DA-based coordination polymer nanoparticles as stable colloidal suspensions with FBS or BSA and high encapsulation yields (close to 60%). The encapsulated DA exhibited a reduced toxicity against DArgic cells by comparison with free DA and a decreased ROS production even with the incorporation of iron ions. In fact, the nanoparticles proved safe *in vivo*, with no significant weight loss or toxicity symptoms for the 30-day period after the last injection administration. The nanostructuring as DA-NCPs also favors (in comparison with the incubation of DArgic cells with free DA) enhanced intracellular retention of the neurotransmitter intracellularly released for extended periods of residence. This fact is mainly attributed to both a very effective incorporation of the nanoparticles by DArgic cells mediated by specific endocytic pathways and to slower metabolism kinetics into DA metabolites (*i*.*e*., DOPAC and HVA) due to the restrictions imposed by the DA supramolecular assembly. The i.c.v. infusion of the nanoparticles into the living brain of Sprague–Dawley rats was sufficient to promote an increase in striatal and SNpc DA levels as soon as 2 h after administration. DA metabolites also increased accordingly, suggesting an efficient clearance of the excess of DA in these living tissues.

Finally, we studied direct delivery of the nanoparticles to the brain *via* nasal administration. Our findings showed an efficient delivery of DA to the nigrostriatal pathway capable of attenuating motor alterations in a 6-OHDA-induced animal model of PD. On top of that, the presence of iron ions was used to track the nanoparticles by MRI after postprocessing of the *T*_1w_ and *T*_2w_ images. All in all, these results position this last generation of DA-based nanoparticles in the field of DA replacement therapy with nanoparticles.

## Materials and Methods

### One-Pot Synthesis of DA-NCPs

DA-NCPs were prepared according to our previously published method for the synthesis of NCPs with some modifications.^41,44,79^ Briefly, a mixture of DA hydrochloride (0.5 mmol, 95 mg) and BIX (0.25 mmol, 60 mg) was dissolved in 12.5 mL of ethanol. Under constant magnetic stirring (900 rpm), an ethanolic solution of Fe(CH_3_COO)_2_ (0.25 mmol, 44 mg in 2.5 mL of ethanol) was added to the DA–BIX solution. The reaction mixture was stirred at room temperature for 2 h, and the suspension rapidly turned from colorless to black, which indicates the formation of a fine precipitate. After 2 h, the precipitate was centrifuged at 8000 rpm for 10 min. The pellet obtained was resuspended and washed three times with ethanol. The sample was divided into two batches; in one of them, the solvent was removed and the solid dried under vacuum and fully characterized by UV–vis, FT-IR, ICP-MS, elemental analysis, ^1^H NMR, and HPLC. The remaining batch was kept under solution to avoid problems with aggregation of the sample during the drying procedure and was characterized by DLS, zeta potential, TEM/STEM, and HPLC. For *in vivo* experiments, the final pellet containing the nanoparticles was resuspended in ethanol (to reach a final DA-NCPs concentration of ≥20.0 mg/mL (w/v)) and stored under solution at 4 °C until use.

### Size and Zeta-Potential Measurements

Mean particle size and zeta potential of the DA-NCPs were determined by DLS using a Zetasizer Nano 3600 instrument (Malvern Instruments, UK). Samples of the nanoparticles were dispersed in five different solvents: ethanol absolute, 70% ethanol, PBS, PBS with 30 mg/mL (∼0.5 mM) of BSA, or Opti-MEM culture medium containing 10% heat-inactivated FBS. In order to study the physical stability of DA-NCPs over time, an aliquot of nanoparticles was dispersed in 70% ethanol, PBS–BSA, or culture medium with FBS, and each suspension was analyzed immediately or after 10, 30, 60, and 120 min of incubation at 25 °C. In all cases, the nanoparticles were dispersed at a concentration of 100 μg/mL. Measurements were performed in automatic mode, as an average of 14–24 runs and in triplicate.

### TEM and EDX Analysis

Samples of DA-NCPs were suspended in ethanol, diluted to 200 μg/mL, and placed onto carbon-coated grids. After 2 min, grids were dried carefully and negatively stained with a 2% (w/v) uranyl acetate solution for 2 min. Micrographs were acquired under a Hitachi H-7000 TEM with a 75 kV accelerating voltage. The diameter of the particles was measured using ImageJ software.^80^ For EDX analysis, DA-NCPs were diluted in ethanol, layered over carbon-coated grids, and then preparations washed twice with distilled water. An EDX spectrum of the nonstained nanoparticles was obtained in a Jeol JEM-1400 microscope operating at 120 kV, by collecting X-rays emitted from the local volume probed by the electron beam.

### SEM Analysis

The structural properties (shape, surface morphology, and particle size distribution) of the DA-NCPs were studied by a high-resolution field emission SEM in a Hitachi S-570 SEM. Samples of nanoparticles were diluted in ethanol and placed over an aluminum tape. Following solvent evaporation, samples were coated with platinum under an argon atmosphere by using an Emitech K550 sputter coater (Emitech Ltd., Ashford, Kent, UK). SEM micrographs were acquired at an accelerating voltage of 15 kV under a high-vacuum mode and a distance of the sample of 5 mm.

### UV–Vis Spectroscopy and Spectroscopic DA Quantitation

The absorbance spectra of DA-NCPs, as well as spectra of its polymeric precursors (DA, and BIX), were recorded using a Cary 4000 spectrophotometer (Agilent), in the 200–800 nm wavelength range, and using a matched pair of quartz cuvettes of 1 cm optical path length. Immediately before analysis, samples were diluted in filtered Milli-Q water to a final concentration of 100 μg/mL. For the spectroscopic DA quantitation, 5 mg of dried DA-NCPs was dissolved in 5 mL of 1 M HCl, sonicated until the complete dissolution of the nanoparticles, and diluted up to a concentration of 0.1 mg/mL in 1 M HCl. Standards with different DA concentrations (ranging from 0 to 0.4 mM) were prepared by diluting a stock solution (1 mM) of DA hydrochloride in 1 M HCl solution. The absorbance of the different samples was determined at 278 nm using quartz cuvettes (1 cm optical path length) in a Cary 4000 spectrophotometer (Agilent). The amount of loaded DA was calculated from the calibration curve (adjusted to a linear regression model with *R*^2^ > 0.99) (see Supporting Information, Figure S6). The loading efficiency was determined using the following equation: LE in DA-NCPs (%) = (Weight_DA_)/(Weight_NCPs_) × 100.

### FT-IR Spectroscopy

FT-IR spectra of the nanoparticles and precursors were recorded by transmission FT-IR on a Bruker Tensor 27 (Bruker Optics) spectrophotometer. Pellets were prepared by mixing approximately 1 mg of the test sample with 100 mg of KBr, followed by subjection to high pressure using a manual pellet marker. Each spectrum was acquired between 400 and 4000 cm^–1^ at a spectral resolution of 2 cm^–1^.

### ^1^H NMR Measurements

The chemical composition of DA-NCPs was further investigated by ^1^H NMR. In a typical experiment, 5 mg of dry DA-NCPs were dispersed in deuterated methanol. To ensure complete dissolution of the nanoparticles and the liberation of the ligands by demetalation, two drops of deuterated hydrochloric acid were added to the suspension. After incubation for 30 min at 25 °C, ^1^H NMR spectra were recorded using a Bruker Avance DRX 250 NMR spectrometer equipped with a 5 mm broad band inverse probe-head working at 250 MHz for ^1^H. Chemical shifts were referenced to the trace signals of acetone. The reference sample was prepared by mixing 10 mL of acetone into 0.5 mL of deuterated methanol in a WILMAD coaxial insert tube, which was immersed in the 5 mm NMR sample tube (acetone chemical shift: 2.15 ppm). As a reference, the different ligands (DA, BIX) were dissolved in deuterated methanol, acidified with deuterated hydrochloric acid, and characterized by ^1^H NMR spectroscopy.

### Simultaneous Determination of Monoamines Using HPLC-ECD

An HPLC-ECD method for the simultaneous quantification of monoamines (including DA, DOPAC, and HVA) was adapted from previously described methods.^81^ In brief, analyses were performed using a Chromolith Performance RP-18e (100 mm × 4.6 mm, Merck-Millipore, Darmstadt, Germay) column in an Elite LaChrom system from Hitachi (Tokyo, Japan) coupled to a Coulochem 5100A electrochemical detector from ESA (Chelmsford, MA, USA). The detector was equipped with a 5011 dual-electrode analytical cell with porous graphite electrodes set at +5 and +400 mV (for electrodes 1 and 2, respectively). Before the analysis, the column was pre-equilibrated in the mobile phase containing 99% v/v of a buffered aqueous solution (0.1 M citric acid, 0.05 mM EDTA, 1.2 mM sodium octyl sulfate) with 1% acetonitrile. The final pH of the solution was adjusted to pH = 2.7 with triethylamine. Elution of the monoamines was performed with an isocratic elution of the mobile phase, at 25 °C and a flow rate of 1 mL/min. Before analysis, samples of nanoparticles, culture medium, or cell lysates were centrifuged at 15000*g* for 10 min at 4 °C and diluted in homogenization buffer (0.25 M perchloric acid, 100 μM sodium bisulfite, 250 μM EDTA), and 20 μL of each sample was injected into the HPLC system. Calibration curves of DA, DOPAC, HVA, l-DOPA, NA, DHBA, 5HIIA, 3-MT, and 5-HT were prepared by injecting different concentrations from 50 to 1000 pg/mL. All samples and standards were prepared and analyzed in triplicate. The amount of each monoamine present in the samples was calculated from the corresponding calibration curve adjusted to a linear regression model (in all cases, with *R*^2^ > 0.99). Instrument control, data acquisition, and monoamine determinations were carried out using EZChrom Elite version 3.1.7 software.

### Cell Culture

The human BE(2)-M17 (ATCC CRL-2267) and HeLa (ATCC CCL-2) cell lines were maintained in Opti-MEM or minimum essential medium α (MEM-α) medium, respectively. Both media were further supplemented with 10% (v/v) FBS. Cells were grown under a highly humidified atmosphere of 95% air with 5% CO_2_ at 37 °C.

### *In Vitro* Cytotoxicity Experiments

The cytotoxicity of the free ligands (DA, BIX, and iron acetate) and DA-NCPs toward BE(2)-M17 and HeLa cells was evaluated by a resazurin-based assay using the PrestoBlue cell viability reagent. Briefly, BE(2)-M17 cells were seeded in 96-well plates at a concentration of 3 × 10^3^ cells per well and incubated for 24 h. After incubation, cells were treated with DA, BIX, iron acetate, and DA-NCPs at different concentrations (ranging from 0 to 100 μg/mL (w/v) for BIX and iron acetate or from 0 to 250 μg/mL (w/v) for DA and DA-NCP). After 48 h of incubation, aliquots of 10 μL of the PrestoBlue cell viability reagent solution were added to each well and cells were incubated for 1 h at 37 °C in the absence of light and in the presence of a highly humidified atmosphere of 95% air with 5% CO_2_. Fluorescence (ex/em = 531/572 nm) of each well was measured using a fluorescence microplate reader (PerkinElmer Victor 3V). Cell cytotoxicity was evaluated in terms of BE(2)-M17 cell growth inhibition in treated samples and expressed as percent of the control assay. All cytotoxicity experiments were performed at least in triplicate.

### Reactive Oxygen Species Determination

For ROS determination, BE(2)-M17 cells were seeded in a 96-well plate at a density of 10 × 10^4^ cells/well. After 24 h, DCFDA was added at a concentration of 25 μM, and the cells were incubated at 37 °C (5% CO_2_), in the absence of light, for 30 min. DCFDA is a nonfluorescent and permeable dye that, after cleavage and oxidation by ROS or intracellular esterases, generates dichlorofluorescein (DCF), a fluorescent nonpermeable compound. All conditions were tested in the presence or absence of a natural antioxidant, AA, which, additionally, would avoid DA autoxidation. After incubation, the media was replaced, and cells were exposed for 24 h to DA, DA-NCPs (with an equivalent DA concentration of 100, 10, and 1 μg/mL), and iron acetate (at concentrations corresponding to the same as the iron present in DA-NCPs). Hydrogen peroxide (H_2_O_2_) was used as a positive control at a concentration of 250 μM. Cell culture medium was supplemented or not with AA at 20 μg/mL. The fluorescence of each well was measured at 535 nm with a Victor3 microplate reader (PerkinElmer), after excitation at 485 nm. Results are reported as an average of two independent experiments. The analysis of variance was evaluated with one-way ANOVA performed for ROS assays. The comparison between different groups was assessed by applying the Tukey-HSD test. Significance level was set to 0.05.

### Cellular Uptake of DA and DA-NCPs and Metabolism Experiments

To study the uptake and metabolism of free DA and DA-NCPs, human DArgic neuroblastoma BE(2)-M17 cells were seeded at a density of 2.5 × 10^5^ cells/plate onto 60 mm culture plates and grown for 24 h (to reach about 60–70% confluence). After incubation, the medium was removed, and cells were treated with a control medium or with a medium containing the specified concentrations of free DA or DA-NCPs for 2, 6, and 24 h. To prevent DA oxidation during treatments, media were further supplemented with 20 μg/mL AA.

For DA quantification, immediately before cell recovery, aliquots of 90 μL of each culture medium supernatant were collected, mixed with 10 μL of a 10× homogenization solution containing 2.5 M perchloric acid, 1 mM sodium bisulfite, and 2.5 mM EDTA, and sonicated for 10 s on ice. For intracellular monoamine determinations, treated cells were washed three times with cold PBS, scraped, and centrifuged at 1000*g* for 5 min. The resultant pellets were resuspended in a 1× homogenization solution containing 0.25 M perchloric acid, 100 μM sodium bisulfite, and 250 μM EDTA, and samples were sonicated for 15 s on ice. The samples were centrifuged for 40 min at 4 °C. The pellets were dispersed in PBS, and the total amount of protein was determined by the BCA or Bradford protein assay kits (Thermo Fisher Scientific, Waltham, MA, USA). All samples were kept at −20 °C until HPLC-ECD analysis. Uptake rates were determined by measuring the intracellular levels of DA over time. In the case of DA, data were fitted to the following Michaelis–Menten equation: , where *V*_max_ is the maximal velocity as a function of the density of the transporters and *K*_m_ is the value of [DA]_0_ in which the rate of uptake is half of the *V*_max_.

### Preclinical Evaluation

Animal studies were approved by the corresponding local ethics committees, according to the regional and state legislation (protocol CEA-OH-9685/CEEAH-3665). The animals used for the tolerability and MRI experiments were C57BL/6J female mice of 12 weeks of age, weighing 21.5 ± 0.4 g (*n* = 9). These animals were purchased from Charles River Laboratories (l’Abresle, France) and housed in the animal facility of the Universitat Autònoma de Barcelona (UAB). MRI studies were performed at the joint nuclear magnetic resonance facility of the UAB and Centro de Investigación Biomédica en Red – Bioingeniería, Biomateriales y Nanomedicina (CIBER-BBN) (Cerdanyola del Vallès, Spain), Unit 25 of NANBIOSIS, www.nanbiosis.es. The 7T Bruker BioSpec 70/30 USR spectrometer (Bruker BioSpin GmbH, Ettlingen, Germany) equipped with a mini-imaging gradient set (400 mT/m) was used for magnetic resonance acquisitions. The *in vivo* biodistribution and behavioral experiments with Sprague–Dawley rats were performed at the laboratory of animal service within the Vall d’Hebron Research Institute (VHIR).

### Tolerability of Systematically Administered DA-NCPS

Tolerability experiments were done following a previously reported protocol.^82^ In a typical experiment, increasing amounts of DA-NCPs (0.003–0.1 mmol Fe kg^–1^) were intravenously (i.v.) administered (*via* tail vein) to *n* = 3 mice with a 2-day rest period between injections. An additional group of *n* = 3 wild-type (wt) mice was injected with the vehicle as control (PBS with 0.5 mM mouse serum albumin (MSA)). Mice body weight and other welfare parameters were followed for 30 days after the last administration. In these conditions, a dose of 0.1 mmol kg^–1^ of DA-NCPs proved safe, and no significant weight loss or toxicity symptoms were observed during injections and for the 30-day period after the last administration. This value perfectly matches safety administration ranges for related systems such as Fe-based nanoparticles.^83,84^

### Intracerebroventricular Administration of the DA-NCPs

The *in vivo* distribution of the nanoparticles in the nigrostriatal system was assessed in Sprague–Dawley male rats (275–300 g) after intracerebroventricular administration. A permanent cannula (C313G/SPC 3.5 mm, Plastics One) was placed in the right lateral ventricle (AP: −0.8; L: −1.6; DV: −3.6) of the rats under isoflurane anesthesia using a Kopf stereotaxic frame. A dummy cannula was placed in the guide cannula to prevent the entry of material. Three weeks later, DA-NCPs (a total dose equivalent to 50 μg of free DA) were infused into the lateral ventricle with an internal cannula with tubing attached to a 2.5 mL Hamilton syringe placed in a peristaltic pump (KDS 220). A 20 μL amount of a DA-NCP suspension was infused at a 1 μL/min rate. The dummy cannula was placed back into the guide cannula after the administration. Rats were euthanized at 2, 6, and 24 h after the i.c.v. administration of the nanoparticles. The brains were removed, and both right and left ventral midbrains and striata were dissected and kept separately at −80 °C until analysis.

### *In Vitro*, *ex Vivo*, and *in Vivo* MRI Studies with DA-NCPs

For the *in vitro* quantitative magnetic resonance imaging phantoms (MRI-Phantoms), a 7.2 cm inner diameter volume coil was used for excitation and reception of NMR signals. The samples of DA-NCPs were dispersed using agarose (1%) in PBS at pH = 7.4. Five samples at decreasing concentrations (0.6, 0.3, 0.1, 0.05, and 0 mM of metal mL^–1^) were prepared in 1.5 mL Eppendorf tubes. The total volume was adjusted up to 1 mL with agarose in PBS. The suspensions were dispersed in an ultrasound bath just before the solidification of medium and acquisition sequences for assessing longitudinal relaxation (*T*_1_). For *T*_1_ maps, a rapid acquisition with relaxation enhancement (RARE) with variable repetition time (VTR) sequence was used. Echo time (TE) was 7.5 ms acquiring at 18 different repetition times (TR) ranging from 30 to 5000 ms; field of view (FOV), 90 × 60 mm; matrix size (MTX), 128 × 128 (700 μm × 470 μm/pixel); number of averages (NA), 1; RARE factor 2; and total acquisition time (TAT) 21 min 22 s. Parameters for *T*_2_ maps were multislice multiecho sequence (MSME); TR, 3 s; TE ranged from 10 to 600 ms with 10 ms echo spacing; NA, 1; same geometry parameters as in *T*_1_ maps; and TAT, 6 min 24 s.

For the *ex vivopost mortem* MRI studies, a 72 mm inner diameter volume coil was used in combination with a dedicated mouse brain surface receiver coil. The DA-NCPs were dispersed in 2 mL of PBS added with 0.5 mM BSA. The amount finally used for each animal was 5 nmol of metal dissolved in 4 μL of PBS–BSA solution (*n* = 3). The commercial solution of CA-based in GdDTPA (*n* = 3) was used as a standard to compare the enhancement obtained with the NCPs synthesized, and the vehicle was also injected for comparison purposes (*n* = 3). Three injection points were used in each mouse. All administrations were performed *post mortem* using a stereotactic holder, as already described.^76^ High-resolution *T*_1w_ and *T*_2w_ MR images were obtained for each mouse for morphological characterization of the investigated tissue. T_2w_ images (TR/TE_eff_ = 4200/–36 ms) were acquired using a rapid acquisition with relaxation enhancement (RARE) sequence. Parameters for T_2w_ images were RARE factor = 8; FOV, 19.2 × 19.2 mm; MTX, 256 × 256 (75 μm × 75 μm/pixel); NA, 4; TAT, 6 min 43 s. After this, a *T*_1w_ image (TR/TE 350/10 ms) was acquired with an MSME sequence with the same FOV and MTX; NA, 8; TAT, 11 min 56 s. Three ROIs corresponding to the injection coordinates were manually defined after visual inspection in both the area of maximum enhancement and equivalent area of contralateral parenchyma using Paravision 5.1. The RCE—injection site ROI *vs* contralateral parenchyma—obtained in each case was assessed. Only the slice with a better defined contrast-enhanced region was measured. For dual enhancement images (Figure 6c), *T*_1w_ and *T*_2w_ images were processed with the algebraic algorithm (MR signal *T*_1_/MR signal *T*_2_) provided by Paravision 5.1.

To examine the local distribution of DA-NCPs following intranasal administration, *in vivo* MRI was performed using a transmit volume resonator (72 mm inner diameter) for excitation and a dedicated rat brain surface coil as a receiver. Sprague–Dawley male rats (275–300 g) were anesthetized with isoflurane 1–2% in O_2_ at 1 L/min. Breathing (60–80 breaths/min) and body temperature (37–38 °C) were constantly monitored (SA Instruments, Inc., New York, USA) during the MRI experiments. Rats were placed into a holder with bite-bar and ear-bars for optimal head immobilization. A series of *T*_1w_ coronal images and *T*_1_ mapping in the axial plane, covering the nasal cavity from the external naris to the olfactory bulb, were acquired before and immediately after intranasal administration of the nanoparticles. Following the initial MRI scanning, DA-NCPs (40 μL, with a total dose equivalent to 200 μg of free DA dispersed in PBS-MSA) were applied to the right nostril of the animal placed in a supine position by slow administration of drops within 10 min. Immediately after DA-NCP administration, a second MRI scanning was carried out under identical experimental conditions. *T*_1w_ images from seven coronal 1.5 mm slices were acquired with an MSME sequence with TR, 300 ms; TE, 10 ms; FOV, 40 × 35 mm; MTX, 256 × 256 (156 μm × 137 μm/pixel); NA, 4; and TAT, 3 min 50 s. *T*_1_ maps in the axial plane were obtained using a RAREVTR sequence with 7 ms TE and acquisition at 10 different TR values (450, 525, 650, 750, 950, 1200, 1500, 1900, 2500, and 5000 ms). A total of 24 contiguous axial 1 mm slices were acquired with FOV, 35 × 35 mm; MTX, 128 × 128 (273 μm × 273 μm/pixel); RARE factor, 1; and TAT, 32 min 54 s. Data were fitted to an exponential curve using the ISA tool in ParaVision 5.1 in order to estimate *T*_1_ values, as described in the Supporting Information. Mean *T*_1_ values pre- and postadministration of DA-NCPs were obtained from ROIs of three consecutive sections. ROIs were manually defined in areas along the right nasal conduit showing high enhancement on *T*_1w_ images postadministration, and afterward, the equivalent region from the preadministration data set was selected.

### 6-HyroxyDA-Lesioned Rat Model of PD

The 6-OHDA rat model of PD was prepared as described previously.^85^ In brief, for stereotaxic surgery, Sprague–Dawley male rats (250–275 g) were anesthetized with isoflurane (0.5–1.5% in O_2_). The animals were positioned on a stereotaxic frame (Kopf Instruments, Tujunga, CA, USA), and 6-OHDA was unilaterally injected in the right hemisphere of the brain (2 μL of freshly dissolved 6-OHDA hydrochloride prepared with 0.02% ascorbate, Sigma-Aldrich, St. Louis, MO, USA) using a 33-G needle Hamilton syringe (Hamilton Bonaduz AG., Bonaduz, Switzerland). The injection of the neurotoxin was performed into the medial forebrain bundle, with the needle placed 4.0 mm anterior to the interaural line, 1.3 mm lateral to the midline, and 8.4 mm ventral to the surface of the skull, according to the reference coordinates from the Paxinos and Watson rat brain atlas.^86^ After stereotaxic injection, the animals were placed in their cages and monitored until recovery.

### Intranasal Administration of DA-NCPs

For the pharmacokinetic study, 18 healthy Sprague–Dawley rats (275–300 g) were randomly divided into two experimental groups and intranasally administered with vehicle (PBS–MSA solution) or with DA-NCPs dispersed in PBS with 0.25 mM MSA. To perform the intranasal administrations, the animals were anesthetized with isoflurane (0.5–1.5% in O_2_) and put in a supine position. The vehicle (20 μL) or DA-NCPs (20 μL, with a total dose equivalent to 50 μg of free DA) were slowly applied to the right and left nostrils using an Eppendorf pipet. The animals were euthanized at 30 and 120 min after the intranasal administration of the nanoparticles. Then, the brains were removed, and both right and left ventral midbrains and striata were dissected and kept separately at −80 °C until HPLC analysis. To evaluate the performance of DA-NCP administration in comparison with free DA, an additional experiment was performed. In this experiment, nine healthy Sprague–Dawley rats (275–300 g) were randomly divided into three groups and intranasally administered with either vehicle, DA-NCPs, or free DA. These animals were sacrificed 120 min administration postadministration, and the brains dissected as above. The levels of DA present in the samples were determined by HPLC-ECD as described hereafter.

For behavioral testing, 18 6-OHDA-lesioned Sprague–Dawley rats were divided into three treatment groups. The animals were first allowed to habituate to the room and manipulation for at least 1 week before treatments. Two months after the surgery, one group of 6-OHDA-lesioned rats received a semichronic intranasal administration of DA-NCPs at doses equivalent to 800 μg of free DA (four daily doses of 50 μg, between 8 a.m. and 15 p.m.) per day, for 4 days. A second group of PD rats received equivalent doses of free DA. The third group of animals was treated with vehicle (PBS–MSA solution), as a control condition. After semichronic drug administration, rats were subjected to behavioral analyses.

### Apomorphine-Induced Rotation Test

Rats with unilateral 6-OHDA lesions of the nigrostriatal pathway exhibit contralateral rotational behavior when administered with DA agonists such as apomorphine.^85^ This phenomenon can be explained by the hypersensitization of the injured striatum. For assessment of circling behavior, the 6-OHDA-lesioned rats were challenged with 0.5 mg/kg of apomorphine (Sigma-Aldrich, St. Louis, MO, USA) 6 weeks after striatal 6-OHDA injections and after semichronic administration of the nanoparticles. Note that the first evaluation was carried out to determine the effect of the unilateral 6-OHDA lesion on each animal. To perform the rotation tests, rats were introduced inside individual circular cages and connected to an automated rotometer (LE 902 rotameter, Harvard Apparatus, Holliston, MA, USA). The animals were then habituated to the environment for 15 min. Afterward, a subcutaneous injection of apomorphine was given to each of the animals, and the rotational activity was recorded for 1 h. The rotational activity was determined by calculating the total number of net contralateral complete (360°) rotations after subtraction of the contralateral turns. The total duration of the rotational behavior after treatment was also determined for each of the animals and compared with the duration of the rotational behavior before the treatment.

## Supporting Information Available

Details including Materials and Methods, HPLC quantification of bix in the DA-NCPs, DA content of DA-NCPs determined by HPLC, ICP-MS quantification of iron in DA-NCPs, *in vitro* release kinetics under physiological conditions and processing, postprocessing of MR data and encapsulation of EGF; additional tables (Table S1–S4) and figures (Figure S1–S18) (PDF)
