# Supplementary material for: Bioinspired Theranostic Coordination Polymer Nanoparticles for Intranasal Dopamine Replacement in Parkinson’s Disease
Source: ACS Nano. 2021 Apr 22;15(5):8592–609. doi: 10.1021/acsnano.1c00453 (PMC8558863; doi:10.1021/acsnano.1c00453)
Supplement: Supplementary file 1 — nn1c00453_si_001.pdf [file nn1c00453_si_001.pdf]

Supporting Information

Bioinspired Theranostic Coordination Polymer Nanoparticles for Intranasal Dopamine-Replacement in Parkinson's Disease

Javier García-Pardo,[1,2,3] Fernando Novio,[1,4] Fabiana Nador,[1] Ivana Cavaliere,[1] Salvio Suárez-García,[1] Silvia Lope-Piedrafita,[5,6], Ana Paula Candiota,[5,3,2] Jordi Romero-Gimenez,[7] Beatriz Rodríguez-Galván,[7] Jordi Bové,[7] Miquel Vila,[7,8] Julia Lorenzo-Rivera [2,3] and Daniel Ruiz-Molina[1*]*

[1] Catalan Institute of Nanoscience and Nanotechnology (ICN2), CSIC and BIST, 08193 Bellaterra, Barcelona, Spain

[2]Institut de Biotecnologia i de Biomedicina (IBB), Universitat Autònoma de Barcelona, 08193 Bellaterra, Barcelona, Spain

[3]Departament de Bioquímica i Biologia Molecular, Unitat de Bioquímica de Biociències, Edifici C, Universitat Autònoma de Barcelona, 08193 Cerdanyola del Vallès, Spain

[4]Departament de Química, Universitat Autònoma de Barcelona (UAB), Campus UAB. Cerdanyola del Vallès 08193, Barcelona, Spain

[5]Centro de Investigacion Biomédica en Red en Bioingeniería, Biomateriales y Nanomedicina (CIBER-BBN), 08193 Cerdanyola del Vallés, Spain

[6]Servei de Ressonància Magnètica Nuclear, Universitat Autònoma de Barcelona, 08193 Cerdanyola del Vallès, Spain

[7] Neurodegenerative Diseases Research Group, Vall d'Hebron Research Institute (VHIR)-Center for Networked Biomedical Research on Neurodegenerative Diseases (CIBERNED), Edifici Collserola Hospital Universitari Vall d'Hebron, Passeig de la Vall d'Hebron, 129, 08035 Barcelona, Spain

[8]Catalan Institution for Research and Advanced Studies (ICREA), Barcelona, Spain

Table of Content

ACRONYMS

MATERIAL AND METHODS

Materials 4

HPLC Quantification of 1,4-Bis(imidazol-1-ylmethyl)benzene (BIX) in the DA-NCPs 4

DA-content of DA-NCPs Determined by HPLC 5

ICP-MS Quantification of Iron in DA-NCPs 5

In vitro Release Kinetics Under Physiological Conditions 6

Processing and Post-Processing of MR Data 6

Encapsulation of Epidermal Growth Factor (EGF) 8

SUPPORTING TABLES

Table S1. Experimental elemental analysis 9

Table S2. Physical and conformational stability of DA-NCPs 9

Table S3. IC50 values determined for free DA and DA-NCPs against HeLa and (BE)2-M17 cells 9

Table S4. *In vitro* r_1 and r_2 parameters and *ex Vivo* relative contrast enhancement 9

SUPPORTING FIGURES

Figure S1. Dynamic light scattering (DLS) measurements for DA-NPs 10

Figure S2. Mössbauer spectra for DA-NCPs 10

Figure S3. XRD pattern of DA-NCPs 11

Figure S4. FT-IR spectra 11

Figure S5. ^{1}H-NMR spectra 12

Figure S6. Calibration curve for DA concentration determination 12

Figure S7. Encapsulation of recombinant EGF in DA-NCPs 13

Figure S8. *In vitro* release kinetics of dopamine from DA-NCPs 13

Figure S9. Colloidal stability of DA-NCPs in human plasma 14

Figure S10. Effects of free DA and DA-NCPs on BE(2)-M17 dopaminergic cell survival 14

Figure S11. Effects of DA and DA-NCPs on ROS production in BE(2)-M17 dopaminergic cells 15

Figure S12. Effects of iron (II) acetate on ROS production in BE(2)-M17 dopaminergic cells 15

Figure S13. Additional cytotoxicity experiments 16

Figure S14. Extracellular DA levels determined by HPLC-ECD 17

Figure S15. Evolution of the intracellular levels of the DA metabolites DOPAC and HVA 18

Figure S16. Levels of DA, DOPAC, homovanilic acid (HVA), serotonin (5-HT) in the SNpc 19

Figure S17. T_1 and T_2 measurements 20

Figure S18. Intranasal administration of DA-NCPs 21

REFERENCES 22

ACRONYMS

AA: Ascorbic acid

BIX: 1,4-bis(imidazol-1-ylmethyl)benzene

BSA: Bovine Serum Albumin

CA: Contrast agent

Cb: Cerebellum

COMT: Catechol-*O*-methyltransferase

Ctx: Cortex

CytD: Cytochalasin D

DA: Dopamine or 3,4-dihydroxyphenethylamine

DA-NCPs: Dopamine Nanoscale Coordination Polymers

DATs: Dopamine transporters

DCF: Dichlorofluorescein

DCFDA: 2′,7′-Dichlorofluorescein diacetate

DHBA: Dihydroxybenzylamine

DLS: Dynamic Light Scattering

DOPAC: 3,4-dihydroxyphenylacetic acid

EDX: Energy-Dispersive X-Ray analysis

FBS: Fetal Bovine Serum

FT-IR: Fourier Transform Infrared Spectroscopy

GdDTPA: gadolinium diethylenetriaminepentacetate

HPLC: High-Performance Liquid Chromatography

HPLC-ECD: High-Performance Liquid Chromatography with Electrochemical Detection

HVA: Homovanilic acid

i.c.v: intracerebroventricular

i.v.: Intravenously

ICP-MS: Inductively Coupled Plasma-Mass Spectroscopy

L-DOPA: L-3,4-dihydroxyphenylalanine

LE: Loading Efficiency

MAO-B: Monoamine oxidase B

MAOB-I: Monoamine oxidase B inhibitor

MEM-α: Minimum Essential Medium α

MRI: Magnetic Resonance Imaging

MSA: Mouse Serum Albumin

MSME: Multi-Slice Multiecho Sequence

NCPs: Nanoscale Coordination Polymer

NM: Neuromelanin

NR: Number of Repetitions

NS-TEM: Negative-Staining Transmission Electron Microscopy

Nys: Nystatin

PBS: Phosphate-Buffered Saline

PD: Parkinson's disease

PDA: Polydopamine

PDI: *Polydispersity Index*

PLGA: poly lactic-co-glycolic acid

PXRD: Powder X-Ray Diffraction

RARE: Rapid Acquisition with Relaxation Enhancement

RCE: Relative Contrast Enhancement

RCE: Relative Contrast Enhancement

ROIs: Regions of Interest

ROS: Reactive Oxygen Species

SEM: Scanning Electron Microscopy

SNpc: Substantia Nigra pars compacta

Str: Striatum

TEM: Transmission Electron Microscopy

VTR: Variable Repetition Time

wt: Wild type

^{1}H-NMR: Proton *Nuclear Magnetic Resonance*

3-MT: 3-methoxytyramine

5HIIA: 5-hydroxyindoleacetic acid

5-HT: 5-hydroxytryptamine receptors

MATERIALS AND METHODS

Materials

All chemical reagents were purchased from Sigma-Aldrich (unless otherwise specified) and used as received. Solvents were obtained from Scharlab and used as received. Cell culture media and culture media supplements were ordered from Life Technologies. 1,4-Bis(imidazol-1-ylmethyl)benzene (BIX) was synthesized as previously reported.[1] Self-assembled DA-based coordination polymer nanoparticles (DA-NCPs) were prepared under general aseptic conditions to ensure the suitability of the final material for biological experiments. Accordingly, the sterility of the final samples used for cell culture was evaluated following standard microbiological procedures.[2] Cell lines were obtained from the American Type Culture Collection (ATCC).

HPLC Quantification of 1,4-Bis(imidazol-1-ylmethyl)benzene (BIX) in the DA-NCPs

The concentration of BIX present in the DA-NCPs was determined by HPLC-UV using an HPLC Waters 2695 separation module coupled to a Waters 2487 UV-Vis detector (suitable for dual detection). Analyses were performed using the Chromolith® Performance RP-18e (100 mm x 4.6 mm) column. Samples of DA-NCPs were prepared by dissolving 1.5 mg of dried nanoparticles in 0.15 mL of a methanol/HCL mixture (50 µl of HCl were dissolved in 1 mL methanol), and then sonicated for 20 min in an ultrasonic bath until a precipitate is observed. The initial samples were diluted to a final volume of 1.5 mL of Milli-Q water and centrifuged 10 min at 16000 x g. After centrifugation, the supernatants were further diluted in 0.1% (v/v) H_3PO_4 buffer containing 262 mg/L sodium 1-octanesulfonate. Before the analysis, the column was pre-equilibrated with 99% (v/v) buffer A (0.1% (v/v) H_3PO_4 buffer containing 262 mg/L sodium 1-octanesulfonate). Elution started with an isocratic gradient of 99 % (v/v) buffer A for

5 min, followed by gradual increase of buffer B (methanol absolute, HPLC grade) from 1% (v/v) to 40 % (v/v) in 25 min. A calibration curve using BIX as external standard was prepared in triplicate. For this, standard solutions with 0, 10, 60, 110, 160 and 210 µg/mL of BIX were prepared by diluting a stock solution of BIX (960 µg/mL in a methanol/HCl mixture) in Milli-Q water. The methanol concentration of each standard was adjusted to reach a final water/methanol ratio of 90:10 (v/v). The elution of BIX was detected at 280 nm after 24 min chromatography. Results were adjusted to a linear regression model with R2>0.999 in the 10 to 210 µg/mL range.

DA-content of DA-NCPs Determined by HPLC

For DA quantification using HPLC, 5 mg of dried DA-NCPs were dissolved in 5 ml of homogenization solution containing 0.25 M perchloric acid, 100 µM sodium bisulphite, 250 µM EDTA. Samples were sonicated through an ultrasound bath until complete dissolution, further diluted in homogenization buffer up to three different concentrations, and subjected to HPLC-ECD analysis as described in the Methods section (see Simultaneous Determination of Monoamines using HPLC-ECD). The loading efficiency (LE) was determined by using Equation (1), as described for the UV-vis spectroscopic DA quantitation.

ICP-MS Quantification of Iron in DA-NCPs

The total iron present in DA-NCPs was determined by ICP-MS using a Perkin Elmer NexION 300X spectrometer equipped with an S10 autosampler. Samples of dried DA-NCPs nanoparticles were dissolved at a concentration of about 50 µg/mL in concentrated nitric acid solution and incubated at 25 ºC for 2 h. After incubation, samples were diluted (estimated concentration of iron ranging 10-200 ppb) in 0.5 % (v/v) HNO_3 and injected into the instrument using a peristaltic pump from 15 mL tubes placed in the autosampler. Calibration curves of

iron were prepared with five different concentrations (0, 10, 100, 200 and 500 ppb) by diluting certified reference metal ion solutions. The concentration of iron present in samples was calculated from the corresponding calibration curve, previously adjusted to a linear regression model (R_2>0.99).

In vitro Release Kinetics Under Physiological Conditions

In vitro release kinetics of DA from DA-NCPs was evaluated by the dialysis method. Briefly, a dialysis bag (MWCO:3500 Da) containing DA-NCPs dispersed in sterile PBS was adjusted to pH 7.4 or pH 5.1 in the absence or presence of 0.5 mM BSA. To decrease dopamine oxidation during the experiment, all buffers were supplemented with 0.3 mg/mL of ascorbic acid (AA). Each dialysis bag was placed into 50 mL of buffer (pH 7.4 or pH 5.0) and incubated for 24 h at 37 ºC under constant stirring. To determine the release of dopamine from nanoparticles, and diffused through the dialysis bag, aliquots of 250 µL of the external solution were taken from the dialysate at different incubation times. Samples were diluted 50-fold in 1X homogenization solution (0.25 M perchloric acid, 100 µM sodium bisulphite, 250 µM EDTA), sonicated for 10 min, centrifuged 15 min at 16000 x g and stored at 4 ºC until analysis. Dopamine concentration in samples was determined by HPLC-ECD as previously described.

Processing and Post-Processing of MR Data

Processing and post-processing of T_1 and T_2 maps were carried out with Bruker software Paravision (version 5.1) by using the image sequence analysis (ISA) tool package. T_1 and T_2 weighted MRI were analyzed with ImageJ 1.49V (National Institutes of Health, USA).

Three regions of interest (ROIs) were manually defined after visual inspection both in the area of maximum enhancement and equivalent area of contralateral parenchyma. The relative contrast enhancement (RCE) - injection site ROI *vs.* contralateral parenchyma - obtained in

each case was used for calculations (see Equation S1 and S2). Only the slice with a better-defined contrast-enhanced region in the best study (clear detection of individual RCE regions) was used for measurements. Slices of similar anatomical positions were used for T_1 and T_2 RCE measurements.

$$RCE\,(\%)_{exvivo} = \left(\frac{(Si)}{(Sc)}\right) \times 100$$

Equation (**S1**)

Where S (i) is the absolute signal intensity of the “ipsilateral” region with respect to the contrast administration and that visually shows contrast enhancement, and S (c) is the absolute signal intensity of the equivalent contralateral region, which serves as a control and that is defined as “100%”.

T_1 and T_2 values were estimated through different adjustments to map parameters using the following

$$y = A + C * \left(1 - \exp^{(-t/T1)}\right)$$

Equation (**S2**)

where y is the image signal intensity after t, A is the absolute bias, C is the estimated image signal intensity at t = 0 sec, and t stands for the different TR used in the sequence.

$$y = A + C * \exp^{(-t/T2)}$$

Equation (**S3**)

where y is the image signal intensity after t, A is the absolute bias, C is the estimated image signal intensity at t = 0 sec, and t stands for the different TE used in the sequence.

Finally, dual images reflecting both T_1 and T_2 RCE changes were generated with a post-processing algorithm of imaging division (T_{1w} image / T_{2w} image) producing a colored image highlighting dual RCE (figure 6c of the main manuscript).

Encapsulation of Epidermal Growth Factor (EGF)

To quantify the amount of encapsulated EGF, a series of dilutions of pure EGF were analysed by HPLC (Figure S7). The amount of loaded EGF has been calculated as the difference between the amount of EGF present in the initial reaction and the amount of protein determined from the supernatant after the synthesis. The equations used for calculation of the EE and LC are the following equations:

$$EE(\%) = \frac{Weight\ of\ EGF\ in\ DA{-}NCPs}{Weight\ of\ initial\ EGF}$$ Equation (**S4**)

$$LC\ (\mu g/mg) = \frac{Weight\ of\ EGF\ in\ DA{-}NCPs}{Total\ weight\ of\ DA{-}NCPs}$$ Equation (**S5**)

SUPPORTING TABLES

Table S1

Table S1. Experimental elemental analysis and calculated values for the formula $[Fe(DA)_{1.6}(BIX)_{0.5}(AcO)_{0.2}(H_2O)_{1.9}]$

	**C %**	**H %**	**N %**	**Fe % (ICP-MS)**
Calculated	43.71	5.00	8.78	10.02
Experimental	43.70	4.70	8.80	10.20

Table S2

Table S2. Physical and conformational stability of DA-NCPs in different solvents and biocompatible buffers.

Solvent/Buffer	Size (nm) [a]	PDI	ξ (mV)[b]
Water	171 ± 4.20	0.208 ± 0.022	+25.2 ± 1.92
PBS	1370 ± 721.0	0.484 ± 0.054	-13.1 ± 0.53
PBS-BSA	103 ± 16.2	0.198 ± 0.019	-8.32 ± 0.25
Culture medium	107 ± 27.3	0.245 ± 0.011	ND

[a] Diameter of the nanoparticles determined b dynamic light scattering (DLS).
[b] Surface Z-potential. ND, not determined.

Table S3

Table S3. IC50 values determined for free DA and DA-NCPs against HeLa and (BE)2-M17 cells.

Cell type	Treatment	IC50 ± SE (μg/mL)
**(BE)2-M17**	Free Dopamine	117 ± 11.8
	DA-NCPs	177 ± 36.1*
**HeLa**	Free Dopamine	16 ± 6.6
	DA-NCPs	44 ± 10.9*

*p < 0.05, compared to the Free Dopamine control group; Statistical comparison of fits performed with GraphPad Prism software

Table S4

Table S4. *In vitro* r_1 and r_2 parameters and *ex Vivo* relative contrast enhancement (RCE for T_1 and T_2) for the DA-NCPs and comparison with the commercial GdDTPA CA.

	In vitro phantoms				*Ex vivo*			
	Medium	r_1 (mMs^{-1})	r_2 (mMs^{-1})	r_2/r_1	Medium	RCE% (T_1)	RCE% (T_2)	RCE% (T_1/T_2)
DA-NCPs	PBS	5.1 ± 0.6	1.4 ± 0.3	0.3	PBS	262.0 ± 19.0	73.7 ± 12.0	3.6 ± 1.2
GdDTPA	+	4.4 ± 0.7	4.6 ± 0.2	1.1	+	253.1 ± 32.8	74.9 ± 17.6	3.4 ± 1.4
Vehicle	Agarose	-	-	-	BSA	118.6 ± 8.8	122.9 ± 11.5	0.97 ± 0.07

SUPPORTING FIGURES

Figure S1

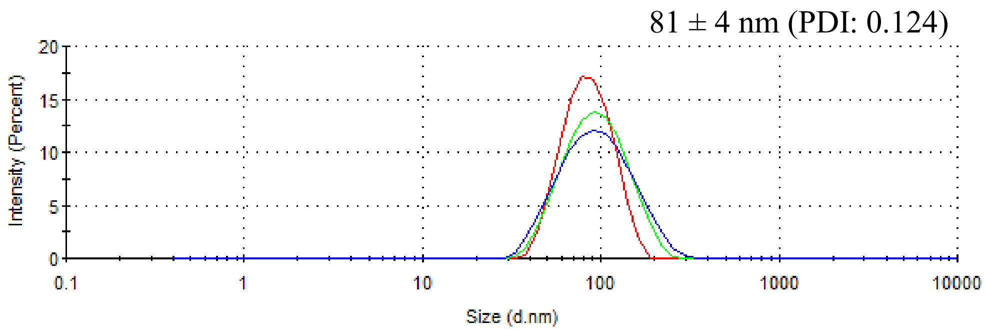

Figure S1. Dynamic light scattering (DLS) measurements of DA-NCPs dispersed in ethanol. The average distribution size is shown with its corresponding deviation after three measurements.

Figure S2

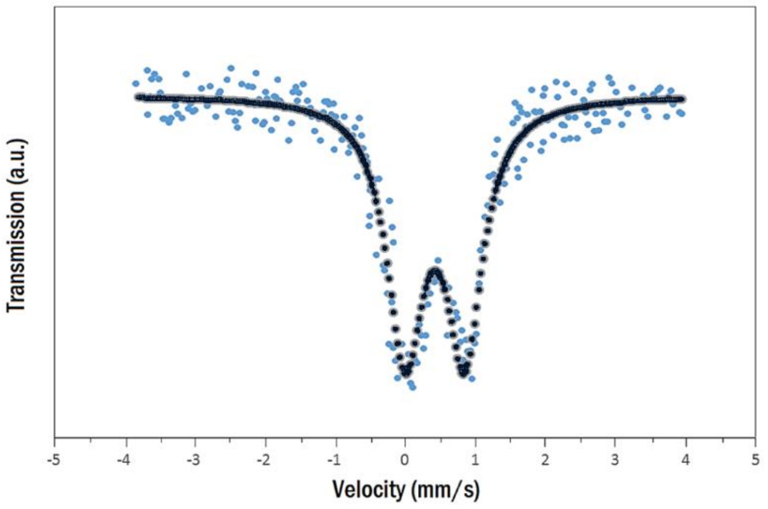

Figure S2. Mössbauer spectra for DA-NCPs at 300 K. Experimental data (small blue dots), and computer fitted spectrum (big grey-black dots) for high-spin Fe(III). Hyperfine parameters of the fitting of the Mössbauer spectra at 300 K showed the isomer shift relative to the metallic iron (δFe), quadrupolar splitting (ΔEq) and the full width at half maximum (Γ). The spectrum was fitted to a single doublet with a ΔEq= 0.85 ± 0.04 mm/s and Γ= 0.28 mm/s. The fitting was centred at an isomeric shift δ= 0.41±0.02 mm/s attributed to high-spin Fe(III) ions.

Figure S3

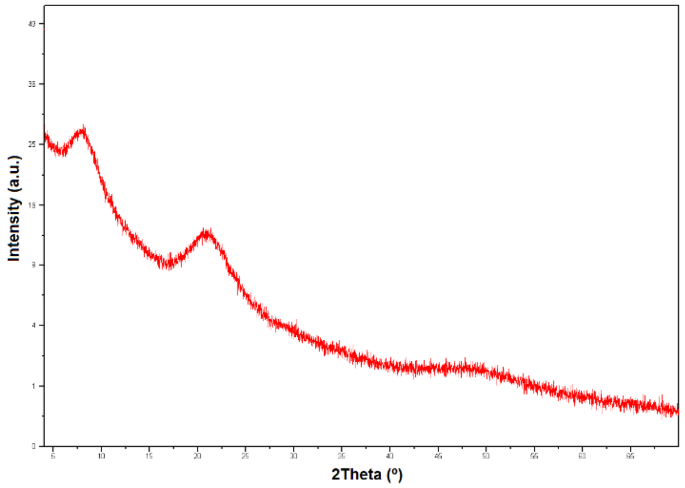

Figure S3. XRD pattern of DA-NCPs

Figure S4

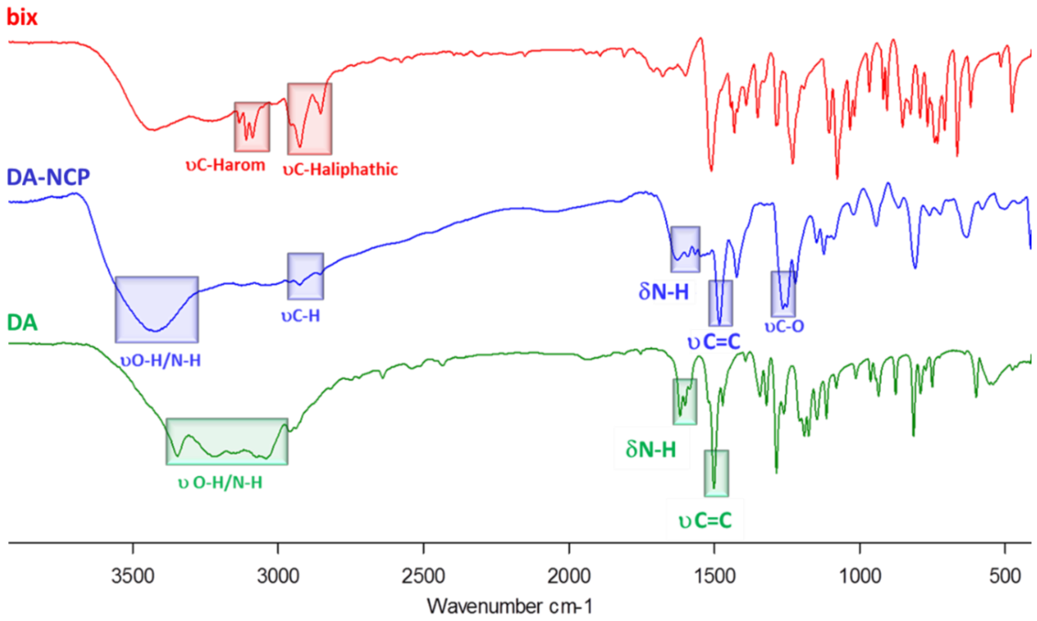

Figure S4. FT-IR spectra of BIX (red solid spectrum), dopamine (DA) (green solid spectrum) and DA-NCPs (blue solid spectrum). Main stretching (υ) and bending (δ) peaks are labeled to facilitate the comparison between spectra. Symbols are: Stretching band of aromatic ring (υC-Harom), stretching band of aliphatic chain (υC-Haliphatic), stretching band of hydroxyl group (υO-H), stretching band of amine group (υN-H), bending band of amine group (δN-H), stretching band of double bounds (υC=C), stretching band of catecholate group (υC-O).

Figure S5

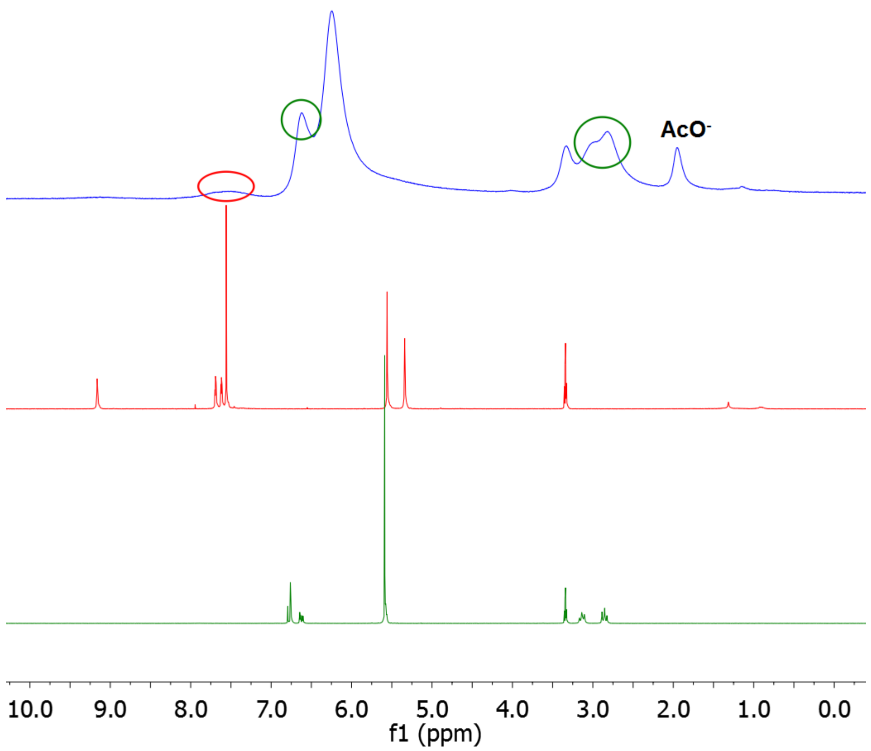

Figure S5. ^{1}H-NMR spectra of BIX (red solid spectrum), DA (green solid spectrum) and DA-NCPs (blue solid spectrum). Circles in the DA-NCPs spectrum show the presence of main contributing signals from BIX and DA, as expected.

Figure S6

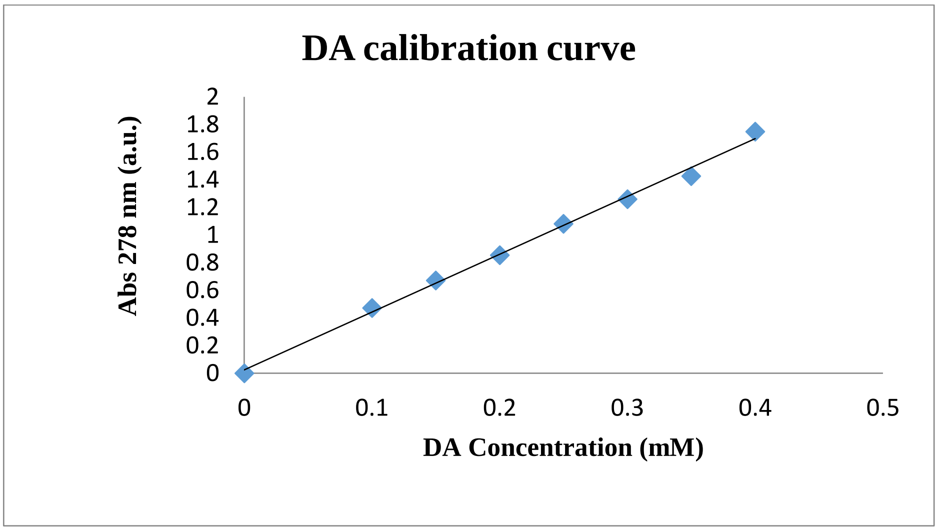

Figure S6. Calibration curve for DA concentration determination using UV-vis spectroscopy.

Figure S7

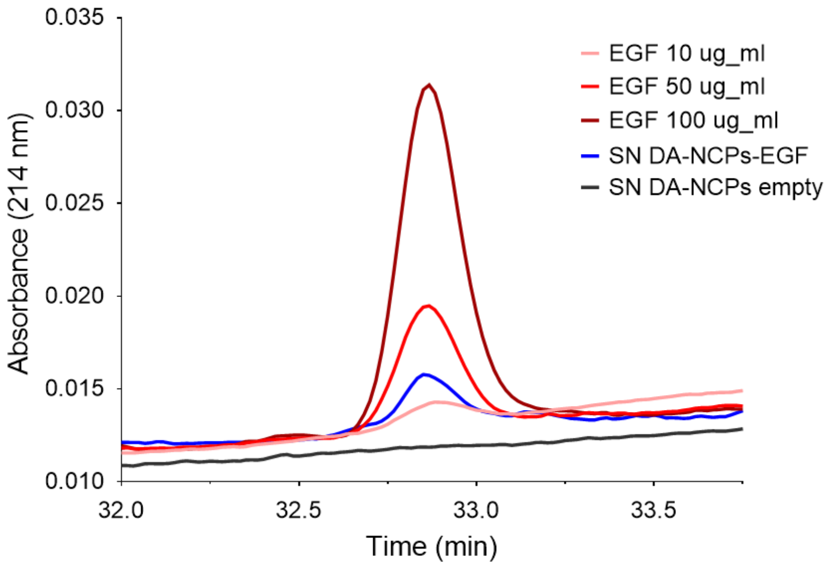

Figure S7: Encapsulation of recombinant EGF in DA-NCPs. Representative HPLC-UV spectra of different EGF concentrations (10, 50 and 100 µg/mL). The supernatant (SN) of the reaction was analyzed for DA-NCPs prepared in the presence (DA-NCPs-EGF) or in the absence of EGF (DA-NCPs empty). For the synthesis of DA-NCPs, 75 µL of EGF at a concentration of 10 µg/µL were mixed with iron acetate solution, which was then quickly added to DA/BIX solution. The reaction was then followed as usual. As control condition, the same reaction was carried out by replacing the volume of EGF solution with PBS. The supernatants of each reaction was collected and lyophilized. These samples were suspended in 1 mL of 0.1 % v/v of trifluoroacetic acid (TFA) water solution, and a dilution of 1:4 in the same buffer was made. The diluted samples, along with different dilutions of EGF as standards, were analyzed by HPLC-UV using a C18 column.

Figure S8

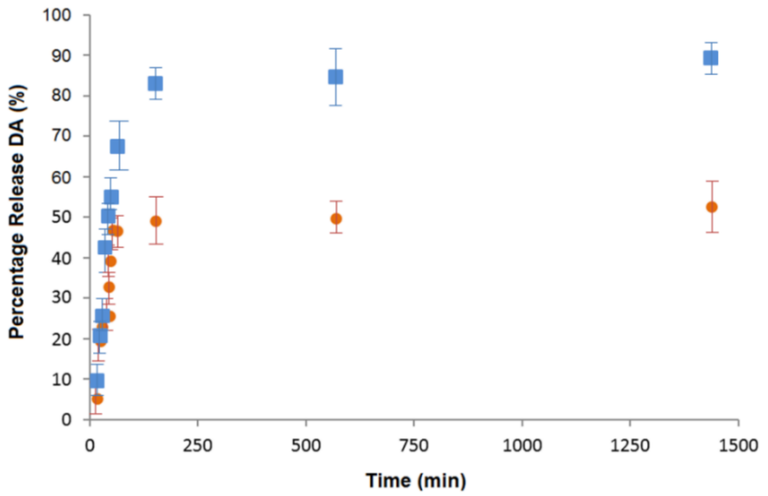

Figure S8. *In vitro* release kinetics of dopamine from DA-NCPs. Experiments were performed in PBS containing 0.5 mM BSA at two different pH values of 7.4 (red dots) and 5.5 (blue squares).

Figure S9

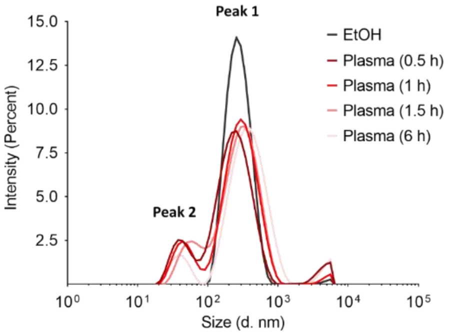

Figure S9: Colloidal stability of DA-NCPs in human plasma. Dynamic light scattering (DLS) measurements of DA-NCPs dispersed in ethanol as reference sample and after incubation of the nanoparticles at 37ºC for different time points (0.5, 1, 1,5 or 6 h) with human plasma. Peak 1; shows the signal corresponding to DA-NCPs. Peak 2; indicates the signal attributable to plasma proteins.

Table S5. PDI values of DA-NCPs in ethanol or plasma at different time points.

Solvent/Buffer	PDI
Ethanol	0.150 ± 0.034
Plasma 0.5 h	0.539 ± 0.011
Plasma 1h	0.530 ± 0.021
Plasma 1.5h	0.534 ± 0.007
Plasma 6 h	0.519 ± 0.032

Figure S10

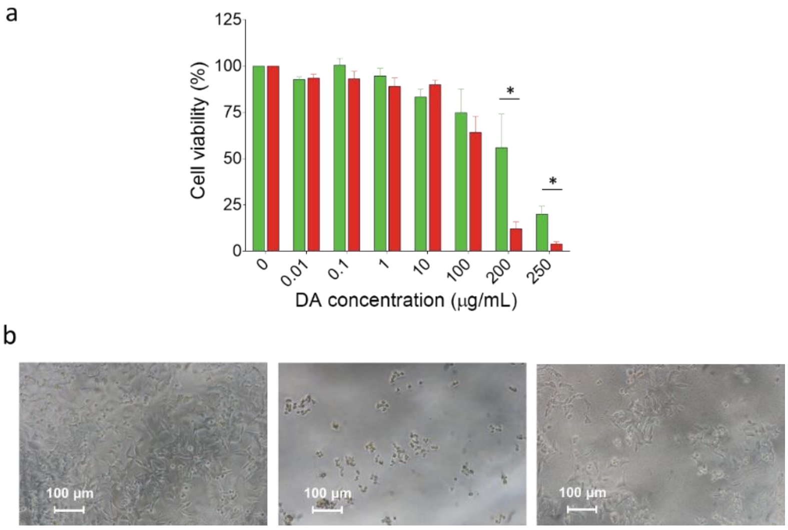

Figure S10. Effect of free DA and DA-NCPs on BE(2)-M17 dopaminergic cell survival. a) Dose-response effect of free DA and DA-NCPs on the viability of dopaminergic cells. To perform the assay, cells were incubated in the absence or the presence of different concentrations of DA (red bars) or DA-NCPs (green bars). After 48 h incubation, cell viability was determined by using a resazurin-based method (see Materials and Methods section for more details). Data are shown as mean ± SEM values (bars). At least three independent experiments were carried out for each concentration. Statistical analyses: ANOVA test was performed to compare DA and DA-NCPs treatments; *$p<0.05$ (n=6). b) Representative optical microscope images showing the effect of control medium (left), free DA (middle) or DA-NCPs (right) at a concentration of 200 µg/mL on the cells, after 48 h.

Figure S11

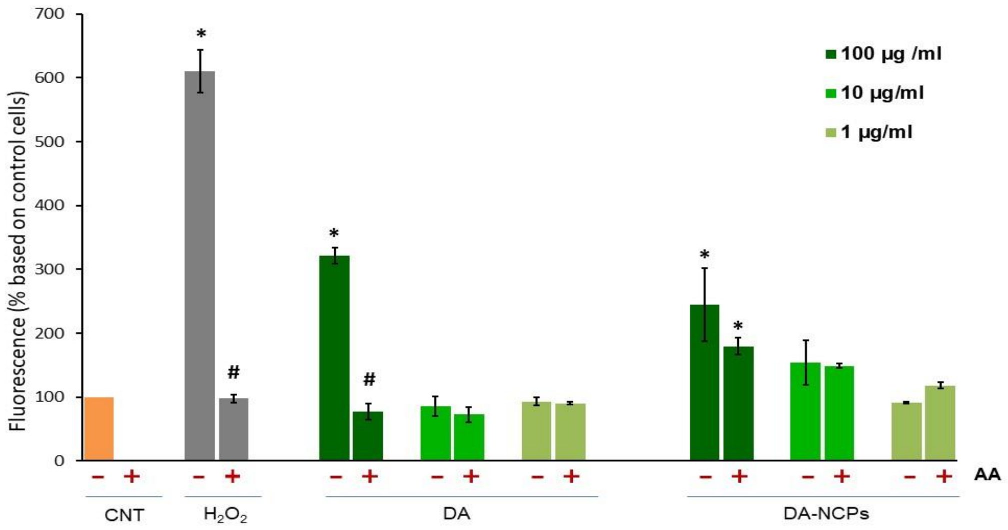

Figure S11. Effects of DA and DA-NCPs on ROS production in BE(2)-M17 dopaminergic cells. Cells were treated with: H_2O_2 as a positive control at a concentration of 250 µM (grey bars), free DA at three different concentrations (100 µg/mL, 10 µg/mL, and 1 µg/mL) and DA-NCPs with equivalent DA concentrations (green bars) for 24 h. Experiments were performed in the presence (+) or absence (-) of AA (20 µg/mL). In all the cases, values are mean ± SEM and n=2. *p < 0.05 compared to the control (CNT), and #p < 0.05, compared to the treatment without AA (one-way ANOVA and Tukey-HSD test).

Figure S12

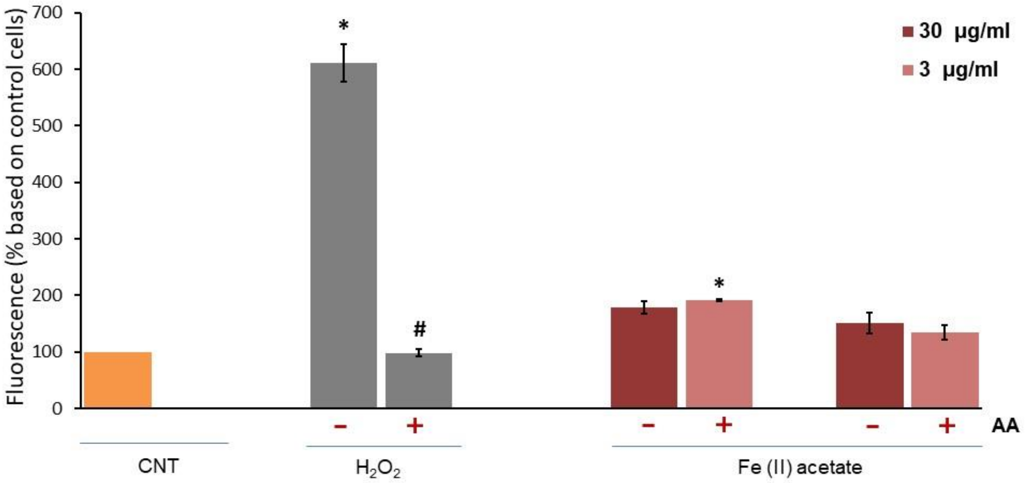

Figure S12. Effects of iron (II) acetate on ROS production in BE(2)-M17 dopaminergic cells, after 24 h treatment at two different concentrations (30 and 3 µg/ml). H_2O_2 (250 µM) was used as a positive control. Treatments were carried out, in the absence (-) or presence (+) of AA (20 µg/mL). In all the cases, values are mean ± SEM and n=2. *p < 0.05 compared to the control (CNT), and #p < 0.05, compared to the treatment without AA (one-way ANOVA and Tukey-HSD test).

Figure S13

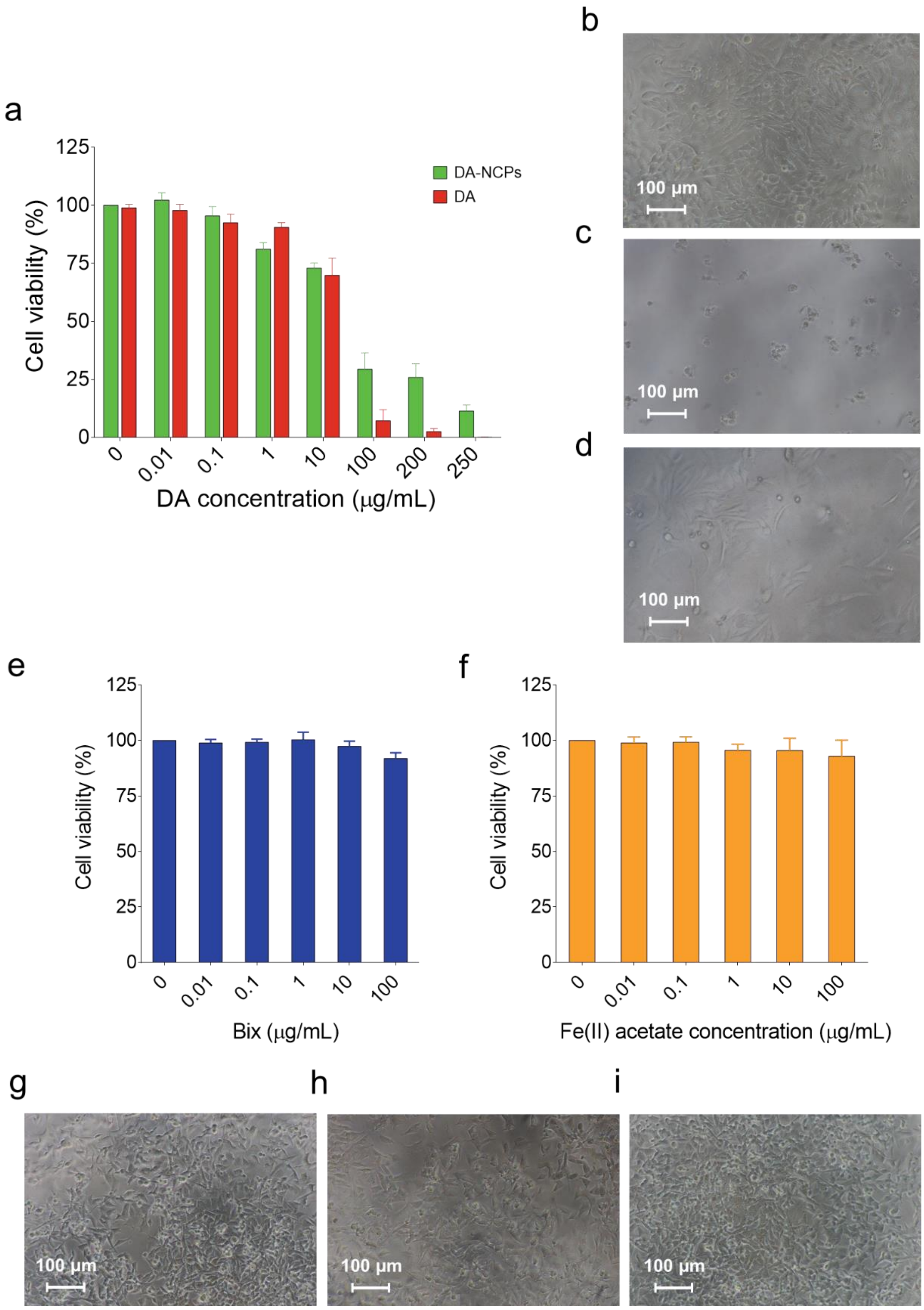

Figure S13. Additional cytotoxicity experiments. a) Effect of free DA and DA-NCPs on the viability of HeLa cells. b-d) Representative optical microscope images showing the effect of b) control medium, and of c) free DA or d) DA-NCPs at a concentration of 200 μg/mL on the cells after 48 h of treatment. In all plots, data are shown as mean ± SE values (bars). e-f) Dose-response effect of e) BIX and f) Fe^{II} acetate on the viability of dopaminergic cells at different concentrations ranging from 0 to 100 μg/mL. Representative optical microscopy images of cells incubated g) in the absence or the presence of h) BIX or i) Fe^{II} acetate at the highest concentration assayed (100 μg/mL).

Figure S14

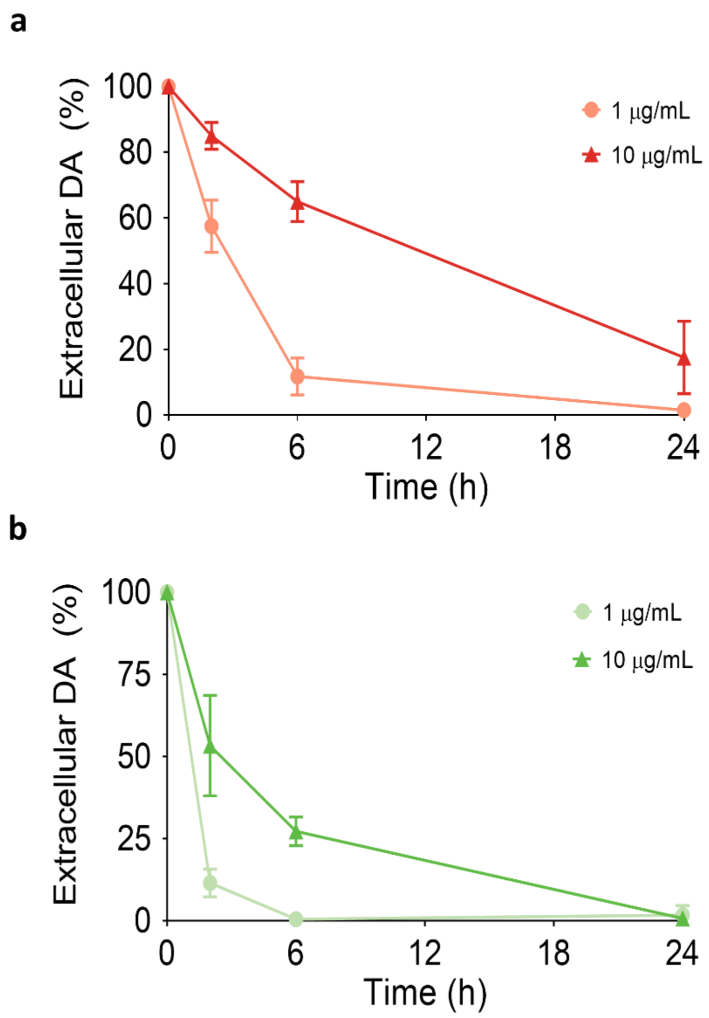

Figure S14. Extracellular DA levels determined by HPLC-ECD after incubation of (BE)2-M17 cells for 2, 6 and 24 h with a) DA or b) DA-NCPs at two different concentrations (equivalent to 1 and 10 µg/mL of DA).

Figure S15

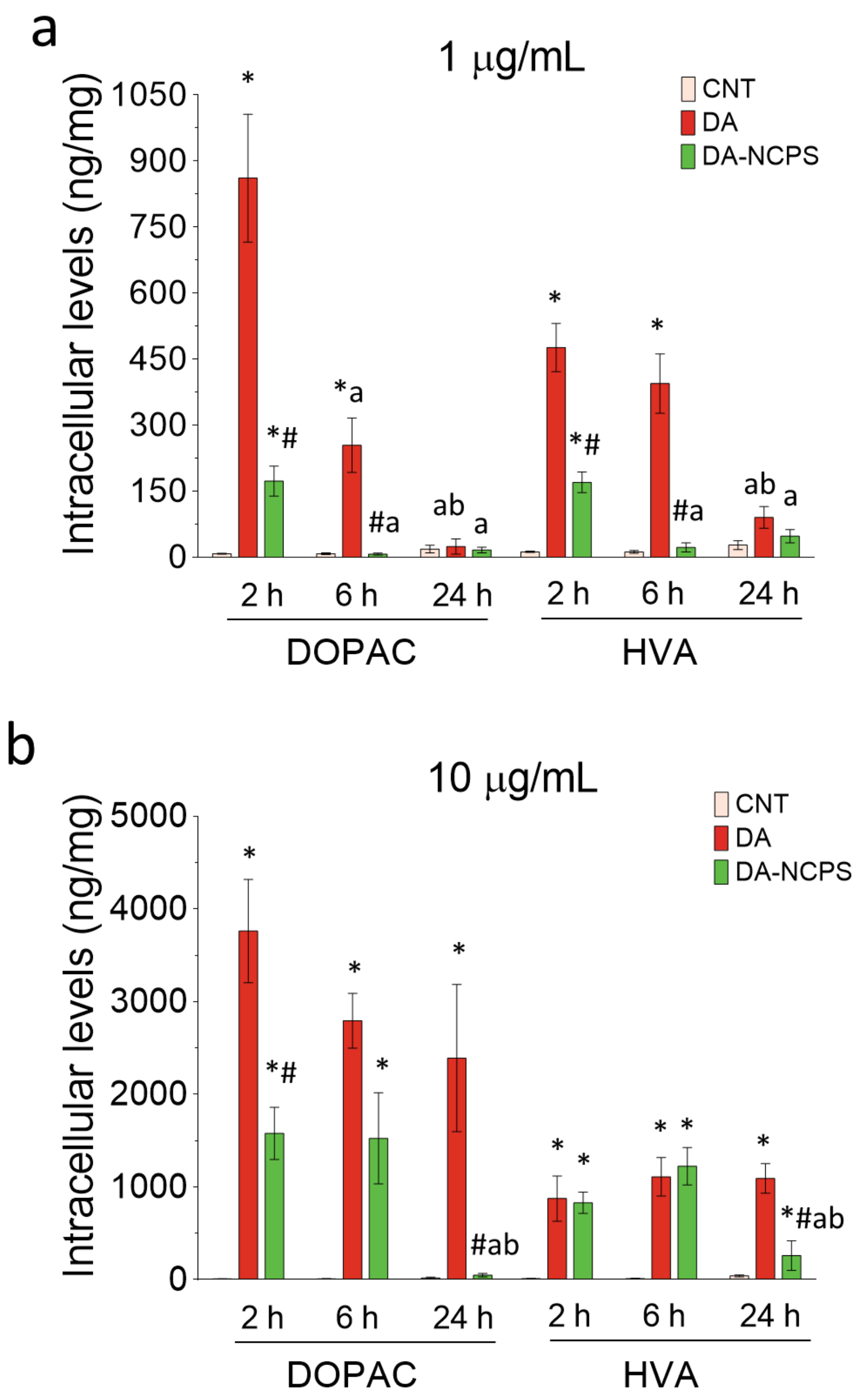

Figure S15. Evolution of the intracellular levels of the DA metabolites DOPAC and HVA over time in free DA- or DA-NCPs-treated cells. Levels measured upon cell treatment at 1 μg/mL of DA or with equivalent concentrations of the nanoparticles. b) Levels determined upon incubation with 10 μg/mL of DA or with equivalent concentrations of DA-NCPs. Values are mean ± SEM. Statistical analysis: three-way ANOVA (full factorial) and Sidak post-hoc correction was applied to the log-transformed data. *p < 0.05, compared to basal levels (0 μg/mL); #p < 0.05, compared to treatment with DA; a p < 0.05, compared to the levels found after 2 h; b p < 0.05, compared to levels found after 6 h;

Figure S16

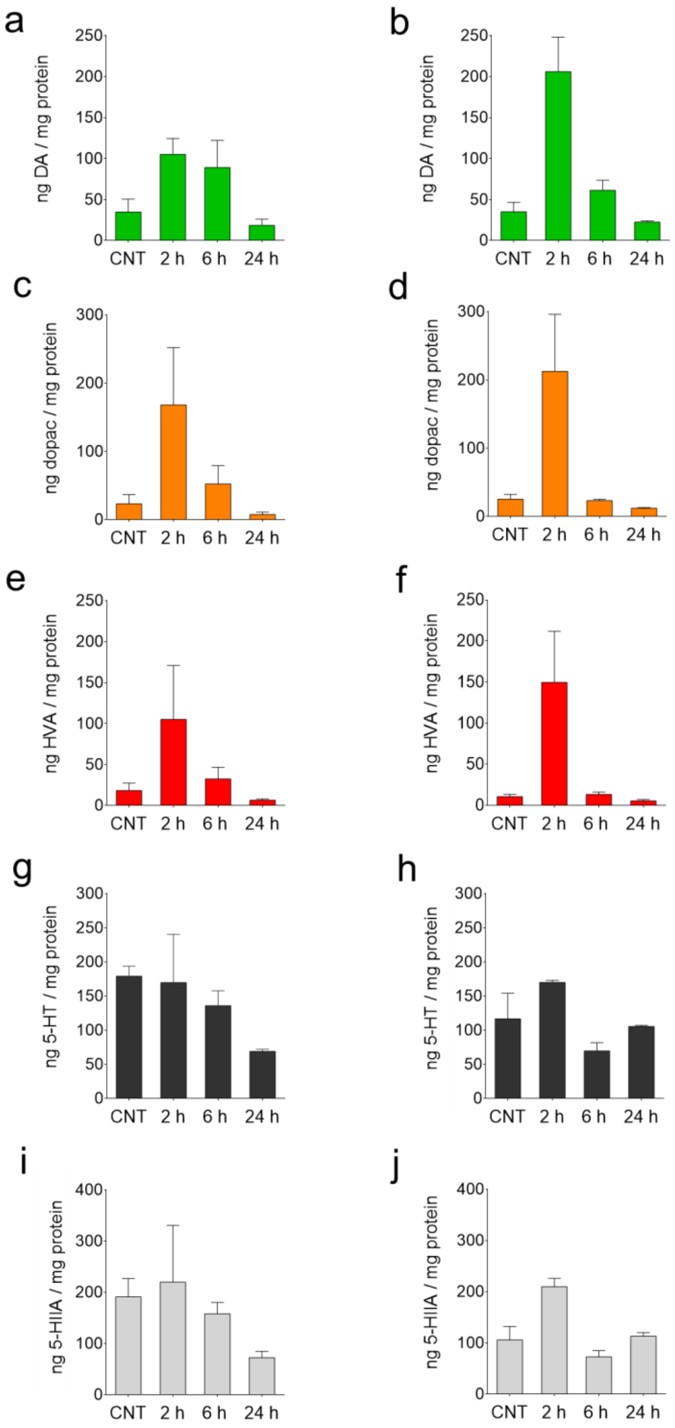

Figure S16. Levels of DA (DA), DOPAC (dopac), homovanilic acid (HVA), serotonin (5-HT) and 5-hydroxyindoleacetic acid (5-HIIA) in the SNpc in the right (panels a, c, e, g and i) and left (panels b, d, f, h and j) brain hemispheres of adult Sprague-Dawley rats administered i.c.v with the DA-NCPs. The nanoparticles were infused in the right ventricle, as shown in Figure 5a. The levels of the different catecholamines were determined by HPLC-ECD at different time points (2, 6 and 24 h) post-DA-NCPs injection. Values are given as mean ± SEM and n=3 independent experiments unless otherwise specified.

Figure S17

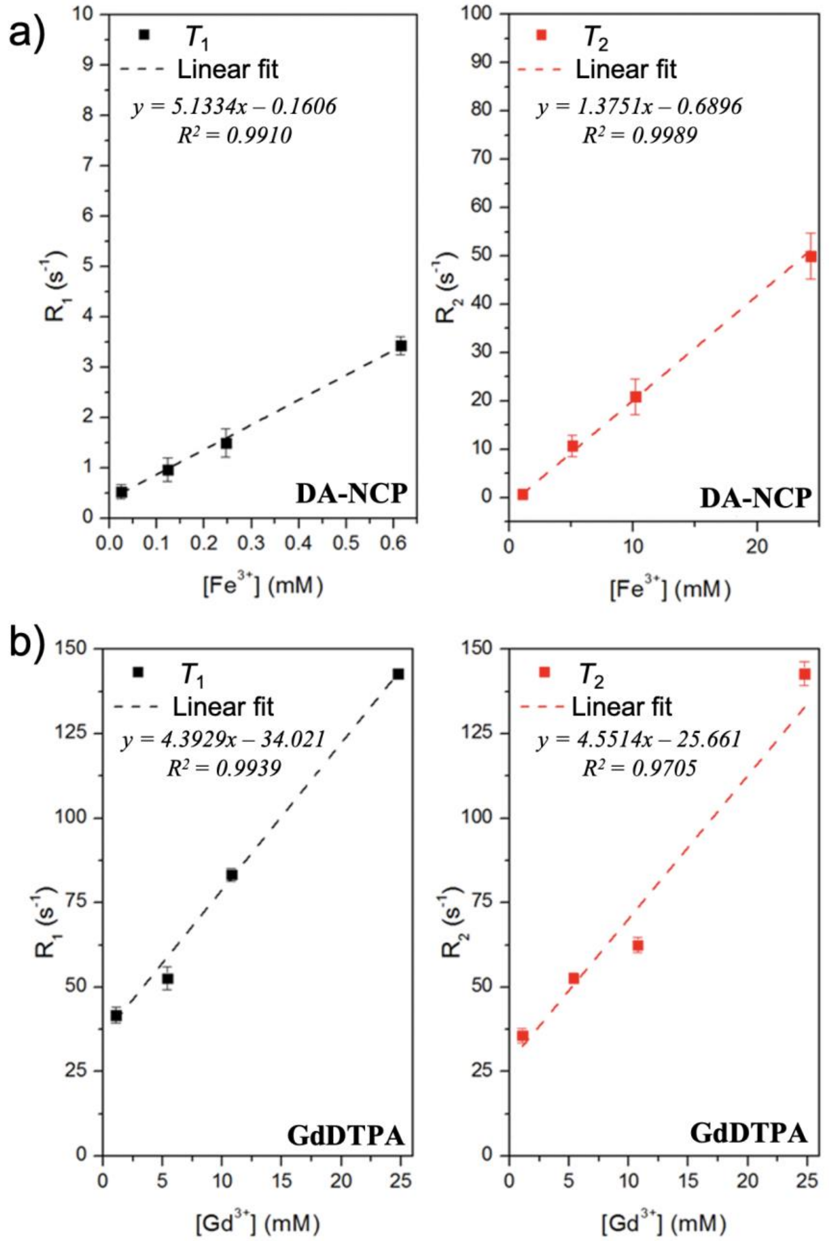

Figure S17. T_1 and T_2 measurements for a) DA-NCPs and b) commercial Gd-based contrast agent (GdDTPA, Magnevist®). Data are shown as mean ± SEM. The experimental data were linearly fitted to obtain the slope of each line (quantitative determination of r_1 and r_2). The results for the DA-NCPs synthesized are shown. From the relaxation times (T_1 and T_2), the relaxivities parameters can be calculated as follow: $Relaxation\ times$: $T_{1,2}$ $[s]$; $Relaxation\ parameters$: $R_{1,2}$ $[s^{-1}]$; $Representation$ $R_{1,2}$ vs $[metal]$; $R_{1,2}/[metal]= r_{1,2}$ ($relaxivity\ parameters$).

Figure S18

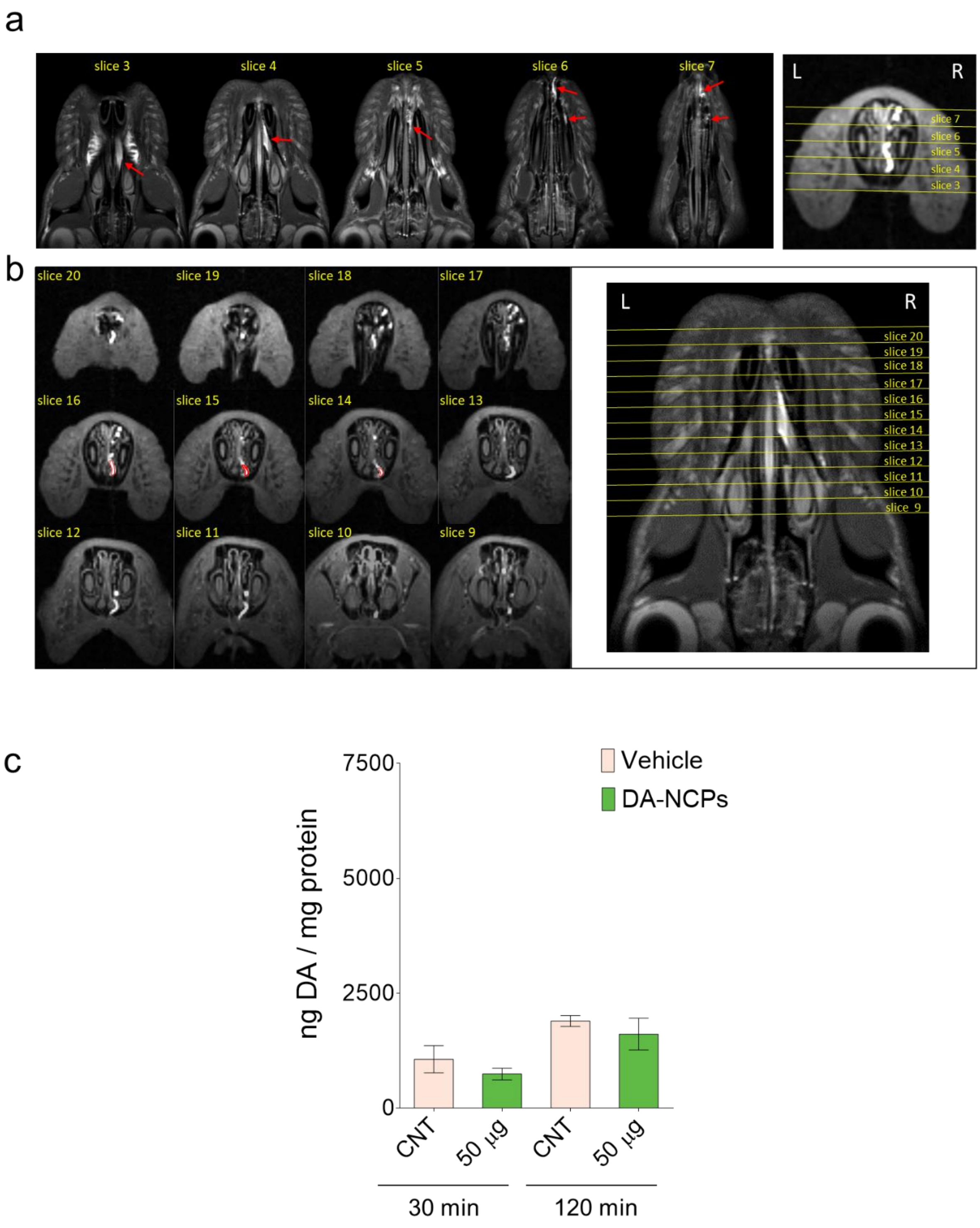

Figure S18. Intranasal administration of DA-NCPs**.** a) Longitudial and b) coronal sections of *in vivo* MRI showing the nasal cavity of a rat after (post-treatment) the intranasal application of the nanoparticles. c) Levels of DA in the left striatum of adult Sprague-Dawley rats administered intranasally with vehicle (light pink) or DA-NCPs (green). The red line in b), demarcates the regions of interest (ROIs) used for the analyses.

REFERENCES

1. Adarsh, N. N.; Novio, F.; Ruiz-Molina, D. Coordination Polymers Built from 1,4-Bis(Imidazol-1-Ylmethyl)benzene: From Crystalline to Amorphous. *Dalton Trans.* **2016,** *45* (28), 11233-11235.
2. Potter, T. M.; Dobrovolskaia, M. A. Analysis of Microbial Contamination in Nanoparticle Formulations. *Methods Mol. Biol.* **2011,** *697*, 131-134.
